# Recent research progress and future directions of intracerebral hemorrhage

**DOI:** 10.3389/fimmu.2026.1845566

**Published:** 2026-07-07

**Authors:** Weidong Liang, Hao Wu, Didi Pan, Yongzhi Wang

**Affiliations:** 1 Department of Neurosurgery, Fuyang People’s Hospital of Anhui Medical University, An Hui, China; 2 Department of Neurosurgery, The Second Affiliated Hospital of Xi ‘an Medical University, Xi’an, China

**Keywords:** intracerebral hemorrhage, damage mechanism, immune response, pathophysiology, signal path, therapeutic targets, future directions

## Abstract

Up to now, Intracerebral hemorrhage (ICH) remains a type of cerebrovascular emergency with high incidence, mortality and disability rates. The secondary brain injury following ICH involves a series of complex pathophysiological mechanisms, including the space-occupying and edema effects of hematoma, abnormal mediation of multiple cell signaling pathways, intense inflammatory storms and immune responses, structural and functional damage to the blood-brain barrier(BBB), progressive increase in intracranial pressure, early hematoma expansion, and neurotoxic effects of blood decomposition product. Over the past few decades, although substantial progress has been made in the construction of risk prediction models, optimization of acute treatment strategies, and improvement of long-term prognosis assessment systems, and dues to the advancement of neuroimaging techniques, improvement in neurocritical care, and innovation in minimally invasive neurosurgery, the overall mortality and disability rates of patients have shown a downward trend. However, it is regrettable that the neurological function recovery of survivors is still generally poor, and the long-term functional improvement rate is very low. Despite extensive and in-depth exploration of the best drug intervention targets and optimal surgical methods for ICH through multiple basic research and clinical trials, these efforts have not yet translated into breakthroughs in clinical treatment outcomes or significant improvements in patient prognosis. This review aims to comprehensively summarize the epidemiological characteristics of spontaneous ICH, the mediation mechanisms of key signaling pathways, the dynamic process of BBB disruption, potential immune-related therapeutic targets, exploration directions of novel drug targets, core mechanisms of secondary brain injury, and key research directions in the future.

## Introduction

ICH is a highly destructive hemorrhagic stroke, mainly caused by spontaneous rupture of intracranial arteries, veins, and capillaries due to non-traumatic factors, resulting in hemorrhage in the brain parenchyma. It has a very high rate of disability and mortality (1). The causes of ICH can be divided into two major types, namely primary ICH and secondary ICH (2). Among them, primary ICH accounts for approximately 80% to 85% of ICH cases, mainly including hypertensive ICH, amyloid angiopathy ICH, and unexplained ICH (3); while secondary ICH mainly includes arteriovenous malformations, aneurysms, cavernous angiomas, arteriovenous fistulas, Moyamoya disease, blood diseases or coagulation dysfunction, intracranial tumors, vasculitis, hemorrhagic cerebral infarction, venous sinus thrombosis, and adverse drug reactions, etc. , which cause ICH (4). Currently, it has become a global public health problem that urgently needs to be addressed (5).

The pathological and physiological processes involved in ICH are extremely complex, including the effects of hematoma’s occupying space and edema, abnormal mediation of multiple cell signaling pathways, intense inflammatory storm and immune response, structural and functional damage of the BBB, malignant increase of intracranial pressure, early expansion of the hematoma, and neurotoxic effects of blood decomposition products, etc. (6).

ICH as a global public health problem that is increasingly severe, the long-term social and family burden it causes is significantly higher than that of ischemic stroke. Multiple epidemiological studies have shown that its disability rate is approximately 50%, and there are significant regional differences. In low-income and middle-income countries with relatively scarce medical technology and resources, the social and family economic burden caused by ICH is particularly significant (7). This poses great challenges for early diagnosis capabilities, timely treatment, and subsequent rehabilitation treatment, and also highlights the complexity of ICH as a highly heterogeneous disease (8).

Epidemiological studies show that ICH has a relatively high incidence rate globally, and its prevalence varies significantly due to factors such as regional distribution, age structure of the population, racial differences, social economic level, genetic factors, and dietary habits (9). This disease not only imposes a heavy burden on patients’ families but also puts a huge pressure on medical institutions (10). Therefore, in-depth exploration and research on the mechanisms of secondary damage involved in ICH after injury and early identification, diagnosis, and intervention are of crucial and far-reaching significance for controlling the deterioration of the condition, improving the treatment of patients’ lives, and restoring long-term neurological function.

**Figure 1:**
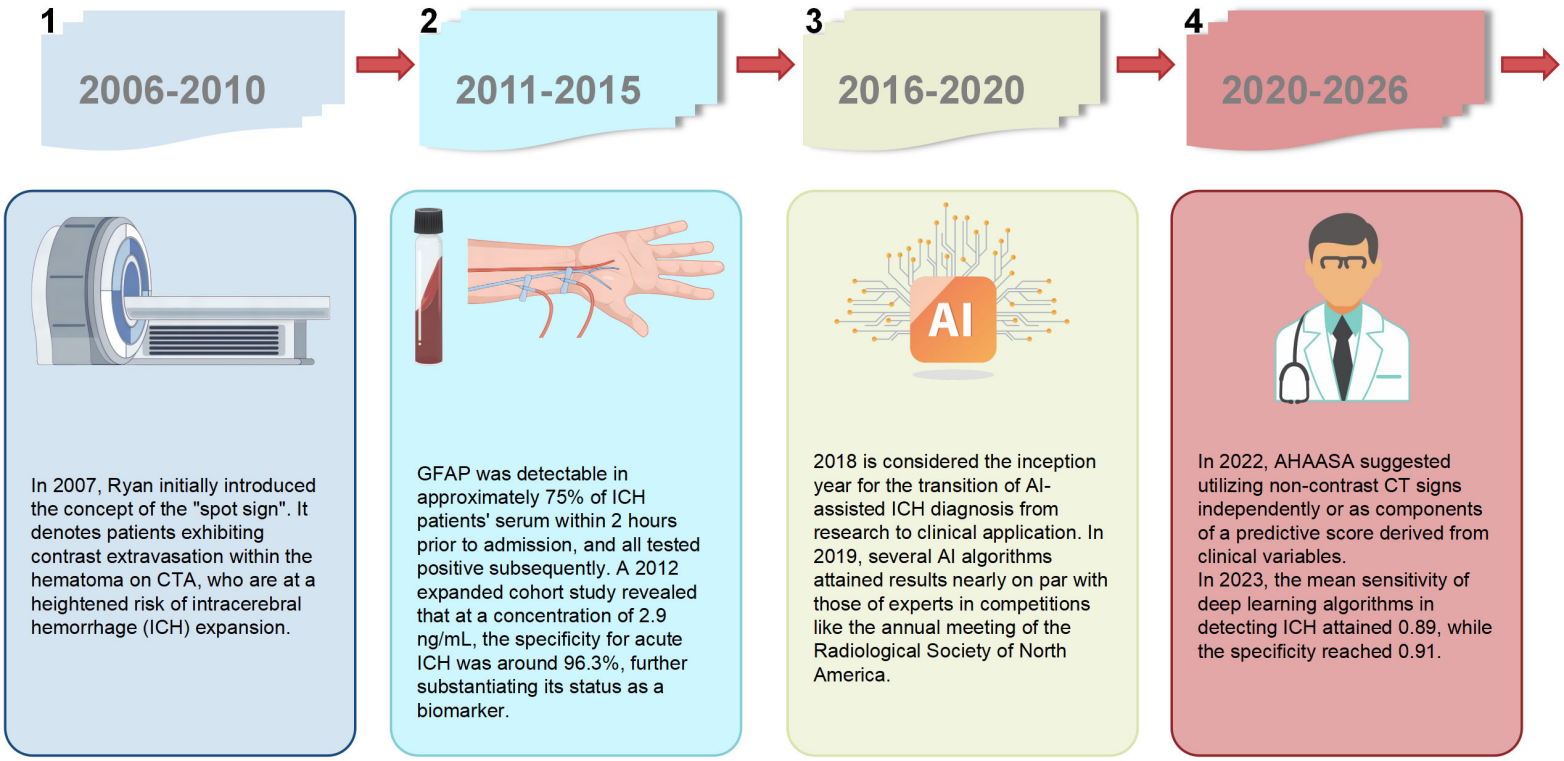
Advances in diagnostic techniques for ICH. In 2007, the “spot sign” was initially proposed as a predictor for the risk of HE. In 2010, the AHA/ASA guidelines were revised to endorse CTA and contrast-enhanced CT for assessing the risk of HE (Class Ilb, Level B evidence). Serum GFAP can be detected in roughly 75% of patients with ICH within 2 hours prior to admission and subsequently in nearly all patients, owing to the abrupt disruption of the BBB and rapid damage to astrocytes induced by acute ICH. Al-driven imaging interpretation technology assumes a pivotal role in stroke diagnosis, facilitating automated ICH detection and differentiation of hemorrhage subtypes. Furthermore, it possesses the capability to identify skull fractures, midline shift, and the mass effect of hematomas. AHA, American Heart Association; Al, artificial intelligence; ASA, American Stroke Association; BBB, blood-brain barrier; CT, computerized tomography; CTA, CT angiography; GFAP, glial fibrillary acidic protein; HE, hematoma expansion; ICH, intracerebral hemorrhage

In recent years, with the advancement of neuroimaging, molecular diagnostic techniques, and the rapid development of Artificial Intelligence (AI), and with a deeper understanding of the pathophysiology and neural repair mechanisms of ICH, the precise diagnosis and individualized treatment of ICH have been further improved (**Figure 1**).

In conclusion, although we have made remarkable progress in risk stratification, cognition, precise diagnostic tools, and treatment strategies for ICH, it remains a clinically highly complex and significantly variable critical disease. Currently, the medical community urgently needs to conduct more systematic and in-depth analyses of the deep pathological physiological mechanisms of ICH, the epidemiological characteristics of different populations, and the heterogeneity of clinical manifestations. Therefore, more in-depth research should focus on further exploring the cascade damage mechanisms after the occurrence of ICH, particularly focusing on the key signaling pathways that mediate inflammatory responses, cell apoptosis, and edema formation. On this basis, efforts should be made to vigorously promote the development of innovative drugs targeting key nodes of these pathways and explore effective strategies to promote BBB repair, neural function remodeling, and the restoration of lost functions, thereby opening new paths for improving the long-term prognosis of patients.

### Epidemiology and economic burdens

**Figure 2:**
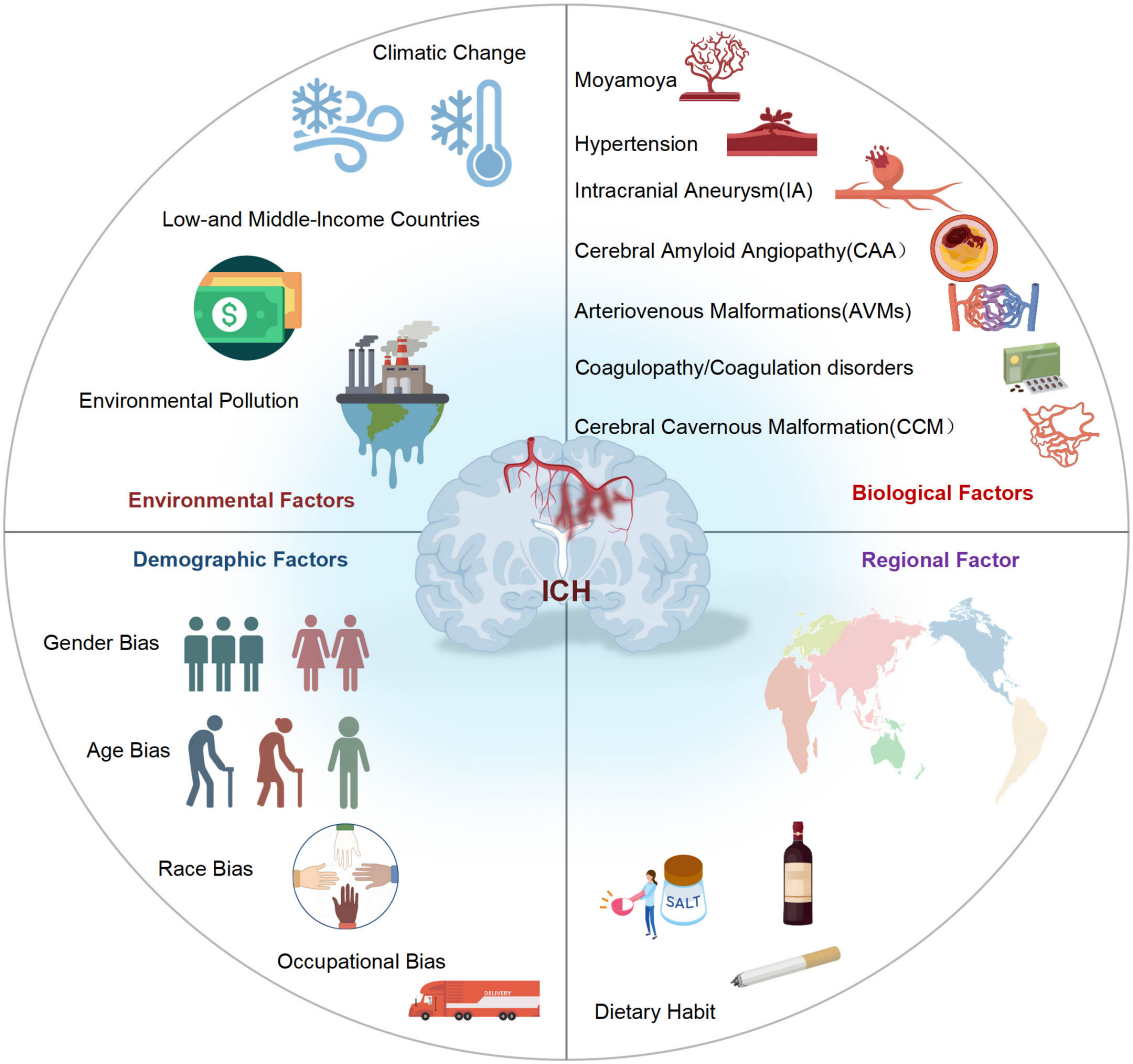
Epidemiological factors for ICH.

Epidemiological factors have a significant impact on the incidence and mortality rate of ICH. According to statistics, among the 12 million stroke patients worldwide five years ago, ICH accounted for approximately 30%; the incidence of ICH is relatively high in some Southeast Asian and African regions (11). From 2004 to 2018, the incidence of primary ICH in the United States increased by approximately 11% (12). In China, the incidence of ICH has decreased slowly in recent years, but it is still significantly higher than that in high-income countries (13). According to incomplete statistics, the incidence of ICH in middle-and low-income countries is approximately twice that of high-income countries, further indicating a positive correlation between the incidence of ICH and the economy and development level of country (14). Currently, studies have found that a variety of factors are closely associated with ICH, including microvascular damage caused by hypertension, atherosclerosis (CAA), and coagulation disorders, etc. , as well as being highly correlated with gender, age, dietary habits, economic level, environmental climate, occupation, and race (1, 14–17) (**Figure 2**). The social and family economic burden caused by cerebral hemorrhage is extremely heavy. In 2021, Fernando, Shannon M et al. from Canada verified that the average hospitalization cost of patients with ICH was approximately 10 times that of other patients (18). In China, the current average hospitalization cost per person has risen to 20, 106 yuan. Compared to 10 years ago, this expenditure has more than doubled (19).

### Pathophysiology and experimental models

**Figure 3:**
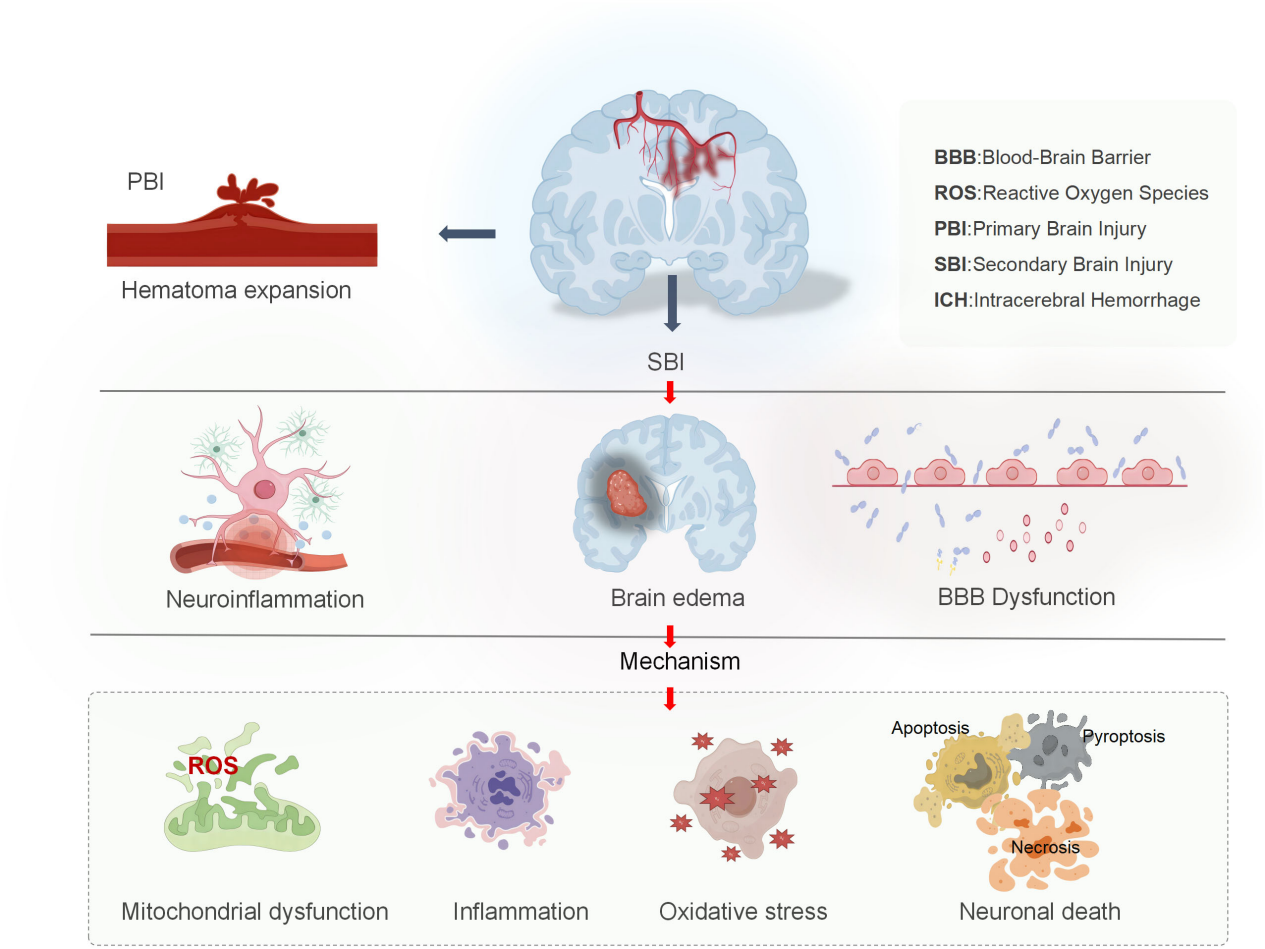
Mechanisms of brain injury in ICH.

Although the location of ICH can vary depending on the specific cause, for instance, hypertensive intracerebral hemorrhage (HICH) often occurs in the basal ganglia region, while amyloid angiopathy is more common in the cerebral lobes, the core pathological mechanism of the neurological functional damage caused by the hemorrhage itself exhibits significant commonalities (**Figure 3**).

The brain tissue damage caused by ICH can be classified into two distinct types:primary injury and secondary injury. Primary injury refers to the physical compression and structural damage to the brain tissue caused directly by the hematoma at the time of hemorrhage; while secondary injury is the further damage resulting from a series of subsequent pathological and physiological reactions on this basis (20). The strategies for alleviating primary brain damage mainly focus on the key goal of preventing cerebral edema, and the core measures include actively controlling blood pressure levels, promptly correcting coagulation disorders, and, when necessary, intervening through surgical procedures (21).

The core mechanism of secondary brain injury lies in the continuous damage to neural functions caused by the toxic components released by the hematoma and their metabolites. Specifically, cytotoxic hemoglobin and its metabolites (such as heme and iron) trigger a strong oxidative stress response by releasing a large number of free radicals, which further causes substantial damage to the brain nerve tissue (22, 23). At the same time, in-depth studies based on the brain hemorrhage model have further revealed that the oxidative stress triggered by these toxic blood metabolites is the key initiating factor for a series of chain pathological reactions. These reactions include significant formation of brain edema, disruption of the structural and functional integrity of the BBB, abnormal activation of the immune system, subsequent neuroinflammatory responses, and ultimately neuronal apoptosis or death (24).

**Figure 4:**
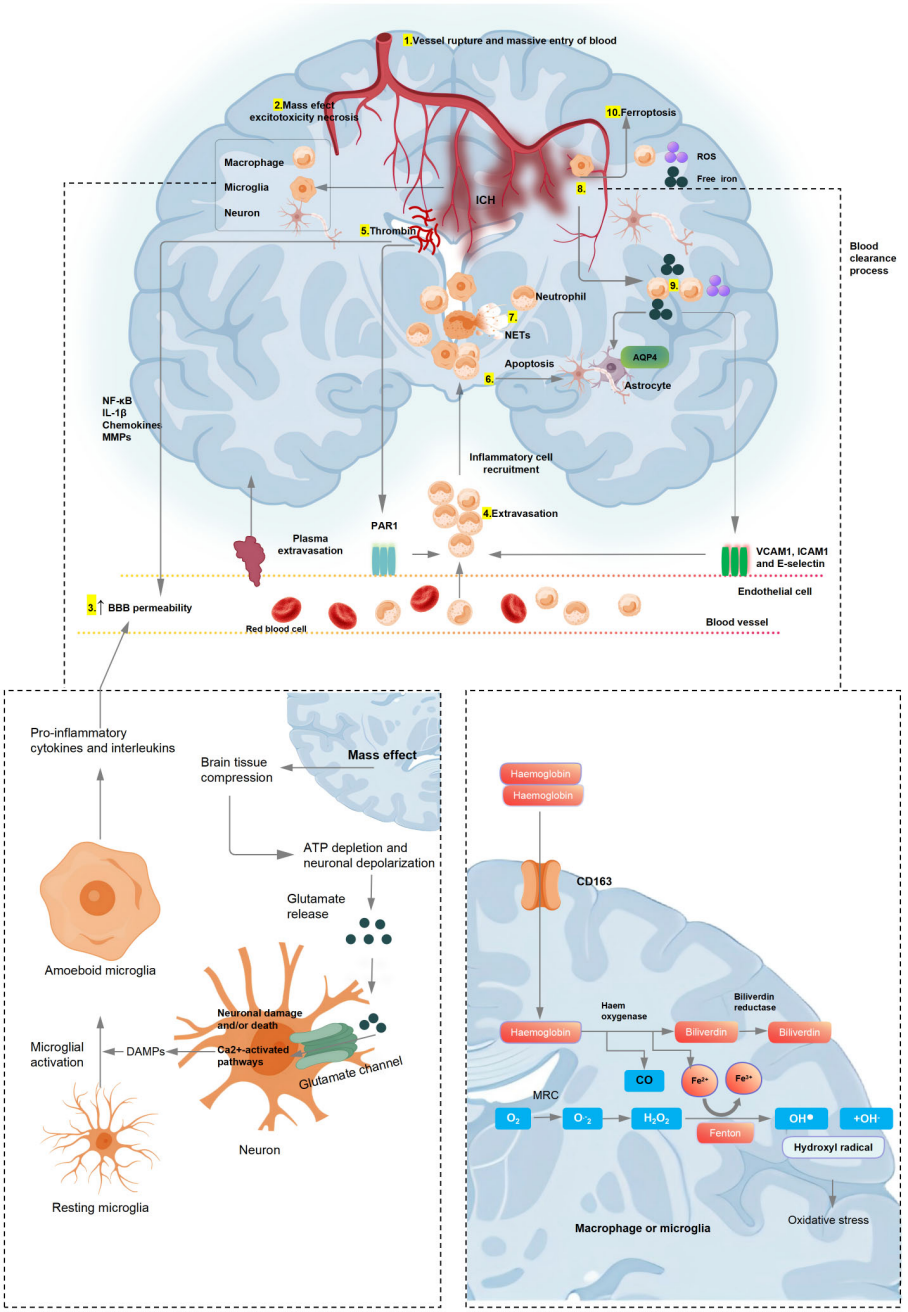
Mechanisms implicated in the pathophysiology of ICH.

Cerebral edema is a key pathological factor leading to secondary neurological function impairment after ICH. Clinical imaging observations have shown that within the first 24 hours after ICH, the volume of the edematous area around the hematoma can increase rapidly by an average of approximately 75%. This rapidly expanding edema band will directly compress the surrounding normal brain tissue, causing a progressive and malignant increase in intracranial pressure, thereby exacerbating cerebral ischemia and secondary damage to nerve cells (25). At the mechanism level, animal experimental models further reveal that the formation of vasogenic cerebral edema is closely related to the activation of thrombin. After ICH, the local coagulation system is rapidly activated, generating a large amount of thrombin. These thrombin not only participate in the hemostasis process but also damage the integrity of microvascular endothelial cells, leading to dysfunction of the BBB and promoting the extravasation of plasma components, thereby forming and aggravating cerebral edema (26, 27). Current research indicates that the concentration differences of thrombin in the nervous system have been clearly confirmed to be closely related to neuronal damage and protective effects. Specifically, high concentrations of thrombin can cause direct damage to neurons and exacerbate the neuroinflammatory process;On the contrary, under pathological conditions such as ischemia or oxidative stress, lower concentrations of thrombin can exert a certain neuroprotective effect by regulating cellular signaling pathways (28). Additionally, The hemorrhagic areas surrounding the brain tissue serve as the source of the injury effect and the core site of the body’s adaptive response. Therefore, their impact on the prognosis of ICH remains unclear (29). Due to the occupying effect of the hemorrhagic mass, the surrounding brain tissue immediately suffers physical damage. Larger hemorrhagic masses can cause an increase in intracranial pressure and brain herniation, which, through mechanisms such as compression and reduced cerebral perfusion pressure, affect the brain tissue far from the hemorrhagic mass. This brain parenchymal area (i. e. , the part of the brain parenchyma at the edge of the hematoma core) is also the vital interface for the body to initiate an adaptive response to ICH, covering processes such as endogenous hematoma clearance, tissue remodeling, and repair (30). The beneficial effects are mainly driven by the sequential transformation of the inflammatory response, which progresses from an inflammatory state to an anti-inflammatory state (31). Although the exact time window for this transformation is not yet clear, it is generally believed to have started within the first week after the occurrence of ICH. This state transition is mediated by cytokines, chemokines, and enzymes released by the white blood cells and glial cells recruited to the injury site. Anti-inflammatory macrophages and microglia can enhance the clearance of hemoglobin, promote hematoma absorption, and limit secondary damage caused by decomposition products of blood. After red blood cells lyse, hemoglobin can bind to hemoglobin-binding proteins, which further bind to scavenger receptors CD163 on the surface of microglia and macrophages;however, during the course of ICH, hemoglobin-binding proteins rapidly deplete, resulting in some free hemoglobin being unable to be effectively cleared, and heme can be released from it (32). Hemosiderin, as a hemoglobin clearance agent, can neutralize its toxicity and mediate its transport to the liver for decomposition metabolism and excretion. The regulatory factors of this clearance system include transcription factor NRF2 and receptor PPARγ (**Figure 4**) (33, 34).

### The formation mechanism of perivascular edema around hematoma (PHE)

Currently, clinical studies have unequivocally demonstrated that perivascular edema around the hematoma (PHE) serves as a crucial and quantifiable indicator of secondary brain injury subsequent to cerebral hemorrhage, and it exhibits a significant correlation with the patient’s unfavorable prognosis (35). Moreover, considering the central position of PHE in the pathological process, the medical community also regards it as a highly promising intervention target for the treatment of cerebral hemorrhage. To facilitate the development of targeted treatment strategies, it is particularly essential and urgent to comprehensively elucidate the relevant formation mechanism of PHE. Although researchers have not yet fully clarified the intricate formation mechanism of PHE, pre-clinical research evidence indicates that the occurrence of PHE at different stages of cerebral hemorrhage is regulated by distinct pathophysiological mechanisms (36). The core pathological basis of PHE is the imbalance of fluid exchange around blood vessels based on the Staline principle (37). In summary, the formation of the osmotic gradient and the elevation of capillary hydrostatic pressure prompt fluid transfer, thereby triggering PHE. Toxic edema and vasogenic edema play significant roles in the occurrence and development of intracerebral hemorrhage (ICH). Toxic edema predominantly occurs in the early stage of ICH and represents an early manifestation of extracellular ion disorder induced by the dysfunction or abnormal activation of ion pumps in endothelial cells and astrocytes. The accumulation of glutamThe area surrounding the hematoma can facilitate the onset of toxic edema (38). The opening of ion channels will intensify the transfer of extracellular water into cells, thereby leading to cell swelling and even cell death (39, 40). Although toxic edema exerts a certain influence on the alterations in water distribution within the brain in the early stage, it cannot genuinely cause swelling of the interstitial space.

**Figure 5:**
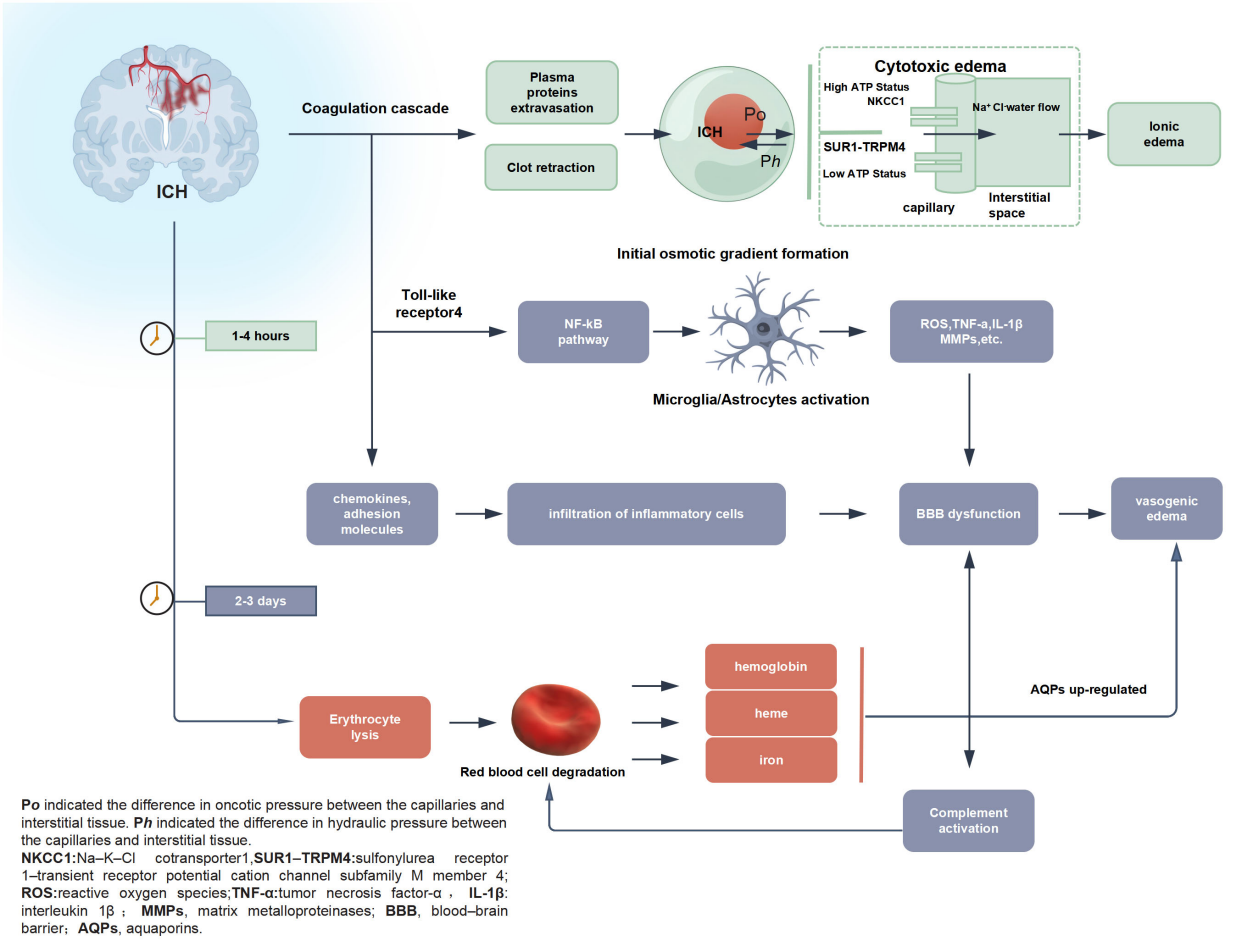
Mechanism of PHE formation.

However, the transmembrane osmotic gradient formed by toxic edema offers the driving force for the occurrence of ionic edema. When toxic edema occurs, water can penetrate into the central nervous system via the aquaporin-4 (AQP-4) expressed in astrocyte foot processes (41). Research has verified that AQP - 4 expression is up - regulated in patients with ischemic stroke and can promote the formation of ionic edema (42). Nevertheless, the mechanism of AQP - 4’s intracellular transmembrane transport remains unclear. Cerebrospinal fluid edema is the primary type of PHE formation in the second stage, characterized by the dysfunction of the BBB, which is triggered by a series of neuroinflammatory reactions. These reactions are associated with mechanical injury of ICH, thrombin activation, and red blood cell lysis toxicity (43). During the immune response related to neuroinflammation, the tight junctions between vascular endothelial cells are disrupted, and through pathways such as attracting inflammatory cells, releasing cytokines and chemokines, up - regulating vascular endothelial growth factor (VEGF) and maMatrix metalloproteinase-9 (MMP-9) further elevates vascular permeability (44). The swelling of endothelial cells and rupture of the cell membrane induced by toxic edema may also augment the permeability of the BBB (45). When the BBB is damaged, the colloid permeability coefficient and hydraulic conductivity coefficient increase, enabling water and macromolecules to more readily traverse the cell membrane and enter the interstitial space of the brain tissue, thereby inducing cerebrospinal fluid edema. Nevertheless, intracranial pressure, blood pressure, and the concentration of permeable molecules in blood vessels also impact the related hydrodynamic and colloid osmotic pressure, thus influencing the formation process of PHE (**Figure 5**) (35, 37).

The inflammatory response subsequent to cerebral hemorrhage is a complex and multi-faceted process (46). In the area surrounding the hematoma, the expression levels of various inflammatory mediators increase, encompassing cytokines (e. g. , pro - inflammatory interleukins (IL)-1, -11, -15, -17, -23, tumor necrosis factor-α, anti - inflammatory IL-4, -10, -23, transforming growth factor β), chemokines (e. g. , CCL2, CXCL8, CXCL2, CXCL5, CCL3, CCL4, CCL20), and matrix metalloproteinases (MMPs) (47–49). Additionally, there are alterations such as microglial cell activation, transformation of brain endothelial cells to an inflammatory phenotype, and infiltration of neutrophils, macrophages, T cells, and natural killer cells. Natural killer (NK) cells infiltrate into the brain tissue (50). The inflammatory response subsequent to cerebral hemorrhage exerts both destructive and protective effects. The former may give rise to adverse outcomes such as early brain injury and brain edema, while the latter facilitates the clearance of thrombi and damaged tissues. The signals that initiate the inflammatory response associated with brain injury stem from the damage - associated molecular patterns (DAMPs) released by damaged parenchymal cells, including high - mobility group protein B1 (HMGB1), S100 protein, adenosine triphosphate (ATP), deoxyribonucleic acid (DNA), ribonucleic acid (RNA), etc. These substances are capable of activating Toll - like receptors on the surface of inflammatory cells. Apart from the damage - associated molecular patterns (DAMPs) released by primary ICH or secondary injury, certain clotting factors generated by cell damage also fall into the category of potent inflammatory mediators. For instance, thrombin produced during hematoma formation, as well as peroxidease 2 (PRX2) and hemoglobin (Hb) released upon red blood cell rupture (51).

In recent years, mitochondrial N-formyl peptide has been verified as a significant DAMP in ICH, which can activate the formyl peptide receptor 1 (FPR1) on the surface of microglia. Research has indicated that inhibiting FPR1 can mitigate the degree of brain edema in ICH mouse models. Simultaneously, the level of N - formyl peptide in the peripheral blood of ICH patients rises and is positively correlated with the The severity of postoperative cerebral edema (52). This suggests that intervention strategies targeting damage-associated molecular patterns (DAMPs) may emerge as a crucial treatment approach for cerebral edema following ICH.

The inflammatory response assumes a pivotal role in the cerebral edema induced by ICH, thereby precipitating vasogenic edema. Subsequent to the occurrence of ICH, the levels of diverse cytokines and chemokines rise in the body, which can disrupt the BBB and ultimately result in vasogenic edema (53).

In recent years, the role of the NOD-like receptor pyrin domain-containing protein 3 (NLRP3) inflammasome in ICH-induced brain injury has garnered extensive attention. NLRP3 can cleave the precursor proteins interleukin (IL)-1β and IL-18 into their active forms. Research has demonstrated that inhibiting NLRP3 can diminish the level of IL-1β in mice and alleviate BBB damage and cerebral edema (47).

Matrix metalloproteinases (MMPs) can also impair the BBB. In fact, collagenase, as a member of the MMPs family, is frequently employed to establish ICH animal models. MMPs can degrade the endothelial basement membrane and tight junction proteins, thereby disrupting the BBB. Tight junction proteins such as desmoglein, claudin-5, and zonula occludens-1 (ZO-1) are substrates of MMP2 and MMP-9. Numerous preclinical studies have investigated the effects of knocking out or inhibiting matrix metalloproteinases (MMP) on cerebral edema after ICH, and some studies have indicated that knocking out MMP-9 and MMP-12 can reduce the extent of edema. The differDiscrepancies in the aforementioned research findings may be associated with the dual function of matrix metalloproteinases (MMP) in the initial injury and the subsequent tissue repair process (54). In the context of cytotoxic edema, neutrophils, macrophages, and microglia can eliminate pathogens through diverse mechanisms, including the release of reactive oxygen and nitrogen free radicals. Nevertheless, these substances possess neurotoxicity and can initiate cytotoxic reactions. Thus, although these cells play a crucial role in clearing hematoma and necrotic tissue following intracerebral hemorrhage (ICH), they may also exert adverse impacts on the surrounding normal cells. Recent investigations on ICH mouse models have revealed that the quantity of natural killer cells in brain tissue significantly increases 12 hours after ICH. These cells exhibit potent cytotoxicity and can intensify the inflammatory response, thereby exacerbating ICH and blood - brain barrier damage (55). In conclusion, the inflammatory response encompasses numerous potential therapeutic targets, some of which have entered clinical trials and demonstrate promising application prospects.

### BBB disruption and brain edema

The disruption of the BBB plays a pivotal role in the formation of perilesional brain edema and can induce vascular edema. The BBB remains intact for several hours subsequent to the occurrence of ICH (56). In pig models, the BBB can preserve the integrity of albumin and large molecules for several hours after ICH (57). The early disruption of the BBB may be attributed to the increased permeability caused By thrombin generated by the hematoma (58). Ruptured erythrocytes release hemoglobin, peroxiredoxin -2 (Prx-2), and carbonic anhydrase 1 (CA -1), which further aggravate the damage to the BBB (59). In fact, the high permeability of the BBB induced by ICH is mediated by multiple mechanisms, including the disruption of endothelial tight junctions and the enhancement of transcellular transport (60). Additionally, within the neurovascular unit, the expression of sulfonylurea receptor 1-transient receptor potential channel 4 (SUR1 - TRPM4) under ischemic conditions facilitates ion entry into the brain, thereby leading to cell swelling and death (61). In ICH models, the expression of SUR1-TRPM4 is up-regulated in the area surrounding the hematoma. Glibenclamide, as a SUR1 inhibitor, can reduce the water content in the brains of young and old rats, improve their neurological function, and decrease the blood - brain barrier permeability in ICH mouse models (46, 62).

During the pathological process of ICH, the damaged neural vascular units (NVUs) trigger the formation and dynamic evolution of edema in the vicinity of the hematoma, and cause the structural and functional integrity of the BBB to be compromised (63). These pathological physiological changes constitute a key link in the process of secondary brain hemorrhage injury. The highly active metabolic activities of the brain require continuous supply of nutrients and energy, and this extensive metabolic demand is maintained by an extremely complex vascular network. This network not only continuously transports essential nutrients and metabolic substrates such as oxygen and glucose, but also efficiently clears the accumulated waste and harmful metabolic products in the brain, and precisely regulates the permeability of peripheral blood components into the brain parenchyma (64). These crucial functions are not accomplished by a single cell, but rely on the extensive, close, and dynamic interactions and direct contacts between various cells of the cerebral vascular system and brain parenchymal cells. To more accurately describe this tight functional coupling relationship, the integrated term “neural vascular unit” has been adopted by the academic community, emphasizing the inseparable functional connection between brain cells and the microvascular system. Under this conceptual framework, the neural vascular unit is defined as a structural and functional assembly composed of neurons, various glial cells, pericytes, extracellular matrix, and vascular endothelial cells. These components work together to maintain the homeostasis of the brain environment, precisely regulate local cerebral blood flow, and can respond coordinately to pathological or physiological stimuli from the outside. Under physiological conditions, each component of the NVU plays an active and specialized role in maintaining this interrelated dynamic balance, so any disturbance in the function or structure of any component will trigger a chain reaction, which will then affect the functional integrity of the entire NVU. What is particularly important is that the NVU constitutes the key structural and functional basis of the BBB; the degradation and destruction process of the BBB after brain hemorrhage, its core mechanism is achieved by directly or indirectly damaging the NVUs and their various components (65).

Pericytes are cells that tightly surround the endothelial cells (BECs) of capillaries and have the function of contraction (66). They are particularly abundant in the brain microvascular system (67). They form a complex interaction network with endothelial cells, astrocytes, microglia cells, and many other types of cells, creating a precise regulatory system that jointly maintains the structural stability and functional integrity of the BBB (68). After the occurrence of ICH, pericytes will abnormally detach from the endothelial basement membrane, resulting in a significant loss of pericytes. The expression levels of their specific markers, such as pericyte-derived platelet-derived growth factor receptor β (PDGFRβ) and NG2 protein, will also decrease significantly. At the same time, a large amount of divalent iron ions (Fe²^+^) will be abnormally accumulated in the pericytes after ICH, and they may die due to excessive oxidative stress (69). This process directly aggravates the damage to the BBB and suggests that inhibiting oxidative stress responses may help improve the survival status of pericytes. Additionally, pericytes are highly sensitive to thrombin produced after ICH. Under the stimulation of thrombin, pericytes will release matrix metalloproteinase-9 (MMP-9), as mentioned earlier, which may further exacerbate the BBB damage after ICH by degrading basement membrane components; however, some studies have also shown that the stimulation of thrombin may instead promote the coverage range of pericytes on blood vessels, demonstrating the dual nature of their response (70). Therefore, similar to astrocytes, pericytes may also be profoundly affected by the immune inflammatory response after ICH, activating related inflammatory signaling pathways and influencing the function of the BBB during this complex pathological process. The precise molecular mechanism of BBB disruption and the specific role of pericytes in this complex pathological process still require further in-depth research to clarify (71).

### Complement system activation and cerebral edema

The early components of the complement system can serve as chemotactic factors, promoting the inflammatory response; whereas the terminal components - the membrane attack complex (MAC) can induce cell lysis (72). In both animal and human studies, the activation of the complement system has been observed (73). C3a and C5a are potent anaphylotoxins, among which C5a is a potent chemotactic factor for polymorphonuclear cells such as neutrophils, monocytes, and macrophages (74). C5a not only stimulates … Neutrophils have their survival time prolonged, and the expression of adhesion molecules is enhanced, thereby facilitating their infiltration into the brain tissue (75). In the research on intracerebral hemorrhage (ICH) mouse models, it was demonstrated that C5a and its receptor commence expression on the first day post - hemorrhage and reach a peak on the third day. Knocking out the C5a receptor in mice can mitigate the activation degree of microglia and the expression level of cytokines in ICH (76). C3a not only exerts the function of promoting the chemotaxis of immune cells but also activates macrophages (77). Further investigations using animal models have indicated that within 2 days after ICH, neutrophil infiltration and astrocyte activation associated with C3 can be detected around the hematoma (78). The activated astrocytes after ICH are also capable of secreting inflammatory cytokines, such as TNF-α and IL-1β (79). C3a and C5a not only induce the degranulation of eosinophils but also release toxic products like myeloperoxidase, ultimately resulting in the generation of reactive oxygen species (ROS) (80). At present, research on C3a and C5a in human intracerebral hemorrhage (ICH) is relatively limited. However, studies based on subarachnoid hemorrhage have revealed that the levels of C3a and C5a in cerebrospinal fluid are on the rise (81). Complement inhibition exhibits a favorable therapeutic effect on brain edema. When combined with the complement activation inhibitor N-acetylglucosamine, it can reduce the formation of brain edema after ICH (82). In studIn studies of the mouse ICH model, it was additionally discovered that C3 receptor antagonists can diminish eosinophil infiltration and cerebral edema, and promote the recovery of neurological function (83). The terminal component membrane attack complex (MAC) may be the primary factor in the formation of cerebral edema, and this conclusion has been verified in animal experiments (84). MAC can create pores on the cell membrane, thereby initiating cell lysis (85). Following ischemic intracerebral hemorrhage (ICH), red blood cells lyse and release pro - inflammatory hemoglobin (Hb), peroxiredoxin 2 (Prx2), and carbonic anhydrase-1 (CA-1) (86). MAC inhibitors, such as glycine ligands, can reduce red blood cell lysis and associated edema on the first and third days post-ICH (87). MAC can also form in neurons, endothelial cells, and glial cells, ultimately resulting in neuronal death and disruption of the BBB. Moreover, MAC itself can induce the production of cytokines and matrix proteins, suggesting that it is a crucial driving factor of the inflammatory response (88).

### Immune characteristics and interactions after ICH

Currently, the therapeutic effect of treating ICH has not achieved satisfactory results. The root cause lies in the fact that the underlying mechanism has not been fully verified. As is well known, inflammation is one of the key factors that cause secondary neurological damage after ICH. During this process, immune cells participate in the neuroinflammatory process, such as microglia, astrocytes, oligodendrocytes, lymphocytes, macrophages, and neutrophilsetc. However, the characteristics of various immune cells and their interactions in the neuro-immune inflammation remain unclear. This section will further explain the immune dialogue after ICH.

**Figure 6:**
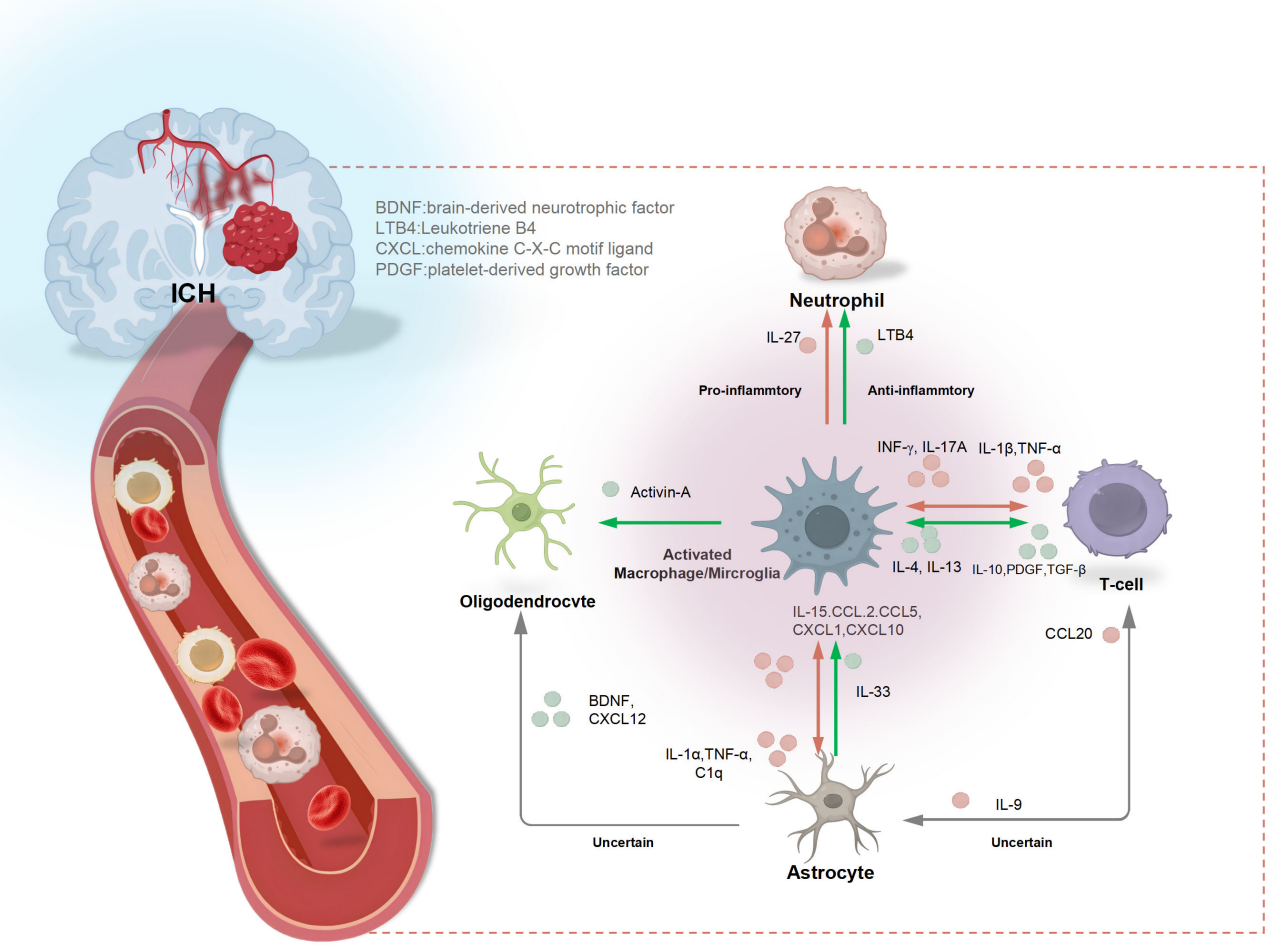
The crosstalk between diverse immune cells after ICH.

A large number of studies have shown that the changes in the inflammatory response after ICH are not solely caused by a single type of immune cell, but are closely related to the interactions between different immune cells (**Figure 6**). In the case of ICH, studies have confirmed that activated microglia can induce astrocytes to transform into type A1 by secreting IL-1α, TNF, and C1q, thereby promoting neuronal apoptosis and exacerbating inflammatory damage (89). The research results indicate that astrocytes expressing IL-15 can promote the polarization of microglia to an inflammatory phenotype. After applying microglia inhibitors, the brain edema condition and neurological function score of GFAPIL-15tg mice with cerebral hemorrhage were significantly improved (90).

The interactions between other immune cells and microglia have also increasingly attracted the attention of researchers. Studies have found that activin A secreted by M2-type microglia/macrophages can promote oligodendrocyte differentiation and promote myelin regeneration, which may become a potential target for treating white matter damage after ICH. In addition, studies *in vitro* and *in vivo* have shown that BDNF produced by astrocytes can promote oligodendrocyte precursor cells (OPCs) to differentiate into oligodendrocytes, which may be an effective target for treating white matter fiber bundle damage after ICH (91). There are also studies that have found that IL-9 produced by helper T cell subsets can accelerate the disease process by inducing microglia to secrete CCL20, promoting Th17 cells to accumulate in the brain tissue (92). Whether these associations occur in ICH and whether there is a certain interaction remain to be further explored in the future.

### Oxidative stress and inflammatory response after ICH

**Figure 7:**
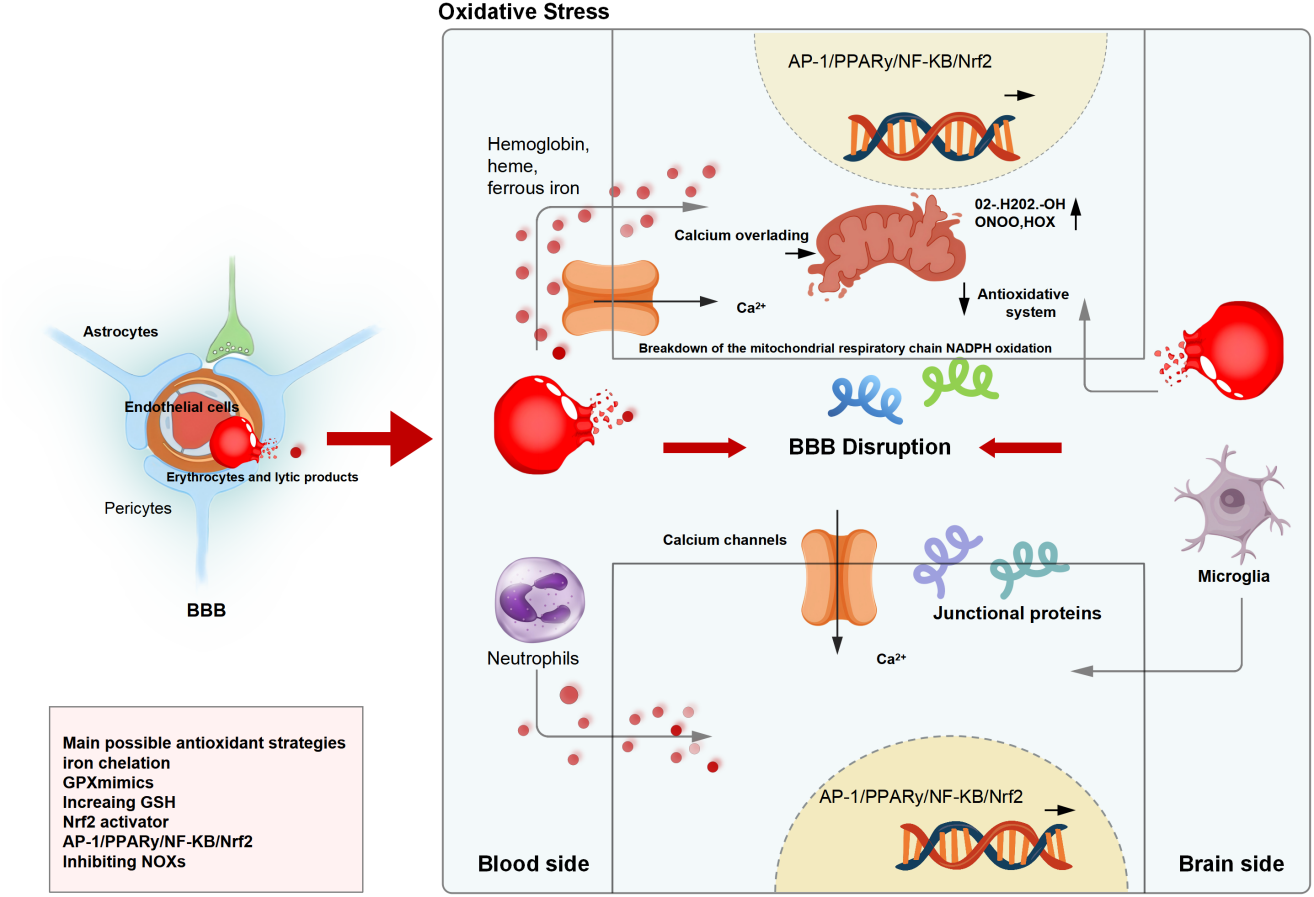
Targeting oxidative stress and inflammatory response for BBB protection in ICH.

After ICH, various components in the blood, such as red blood cells and their metabolites, thrombin and fibrinogen, etc. , will enter the brain parenchyma through the damaged BBB, directly activating and exacerbating oxidative stress and inflammatory responses. And these reactions, in turn, will further damage the structural and functional integrity of the BBB, leading to more blood components infiltrating the brain tissue, thereby forming a continuous amplifying vicious cycle, continuously aggravating brain edema and neurological deficits. A large number of experimental studies and clinical observations have confirmed that BBB disruption plays a key role in the secondary brain injury caused by ICH (**Figure 7**) (93). Therefore, the oxidative stress and inflammatory cascade reaction process triggered by ICH after BBB disruption clearly highlights the crucial importance of protecting the integrity of the BBB as a potential therapeutic target.

In conclusion, future research efforts urgently need to delve deeper into the emerging form of ferroptosis and the complex interactions between oxidative stress and inflammatory responses, as well as the specific molecular mechanisms of BBB disruption after ICH. At the same time, it is necessary to systematically explore the neuroprotective effects and therapeutic potential of various antioxidants and immunomodulators in more rigorously designed clinical trials of ICH.

### Targeting the roles of mitochondria in ICH

**Figure 8:**
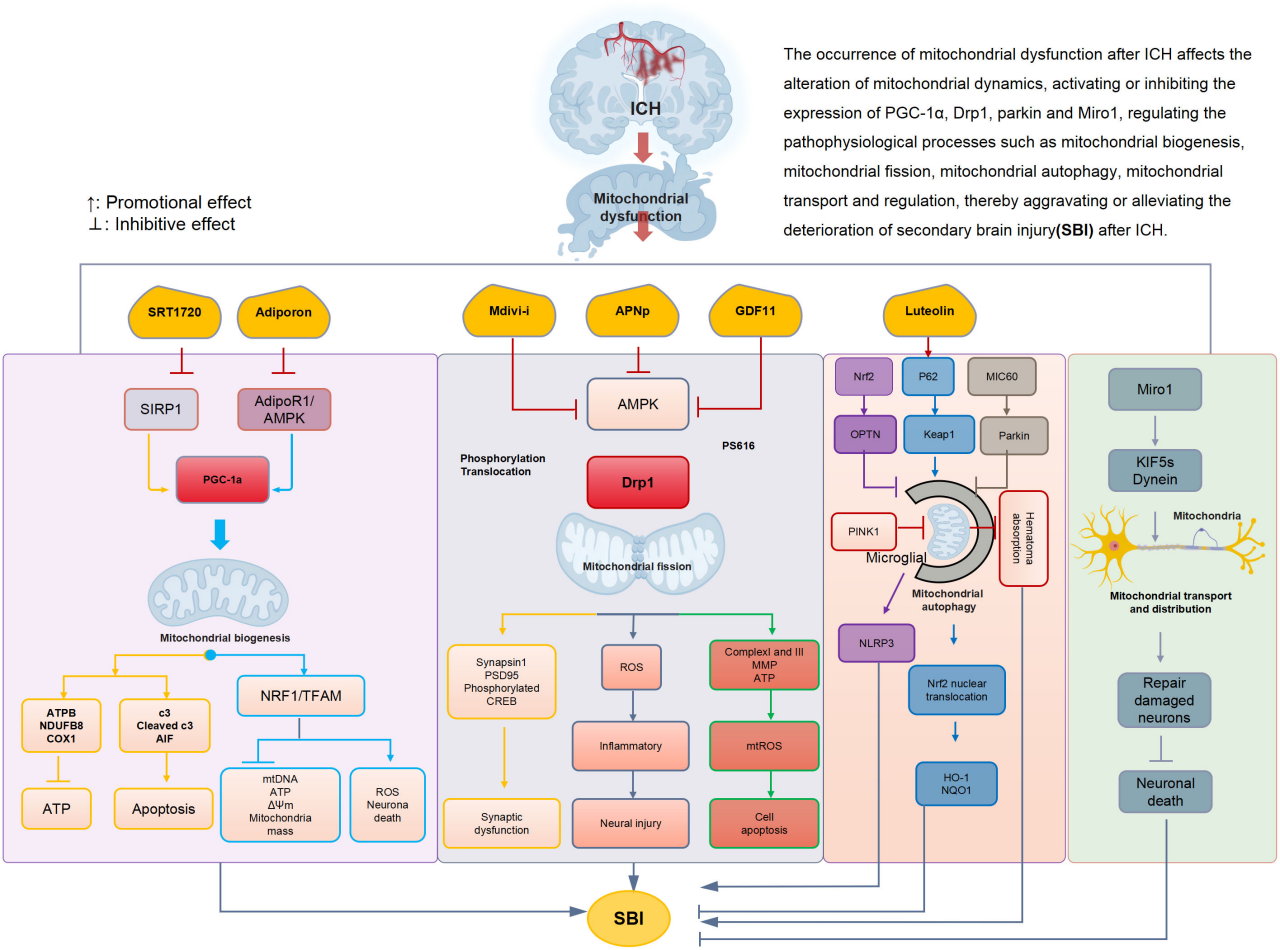
Schematic diagram of mitochondrial dynamics after ICH.

Mitochondria, as the crucial energy factory within cells, primarily undertake the task of producing ATP necessary for cellular activities. These energy molecules provide an indispensable foundation of power for the survival and various physiological functions of cells (94). Beyond their core role in energy metabolism, mitochondria also play a key regulatory role in maintaining calcium ion homeostasis within the cell, by regulating the uptake and release of calcium ions, profoundly influencing cell signaling, metabolic balance, and even cell fate (95). In recent years, the dynamic transport and spatial distribution mechanisms of mitochondria within cells have gradually become a vital focus in the research of central nervous system diseases. Numerous studies have shown that this process is closely related to the pathogenesis of various neurological disorders and is regarded as one of the key links in understanding neurofunctional disorders (96). Specifically, the mitochondrial transport system can precisely deliver mitochondria to regions with high metabolic activity or high physiological demands, thereby effectively maintaining the normal physiological functions and intracellular homeostasis of neurons (97). Studies have shown that the overexpression of Miro1 may reduce neuronal apoptosis after ICH by promoting the transport and spatial distribution of mitochondria mediated by motor proteins in damaged cells, effectively promoting neural function recovery and thereby alleviating common secondary brain injuries after ICH. Particularly, after ICH occurs, mitochondrial function is often significantly affected, with imbalances in key regulatory links such as biosynthesis, fusion and division, autophagy clearance, transport, and distribution, ultimately leading to disordered mitochondrial dynamics (**Figure 8**) (98). This series of dysregulation changes not only significantly alter the number, total biomass, internal morphology, and core functions of mitochondria, but also deeply participate in the complex pathological process of secondary hemorrhagic brain injury.

### Mitochondrial dysfunction after ICH

**Figure 9:**
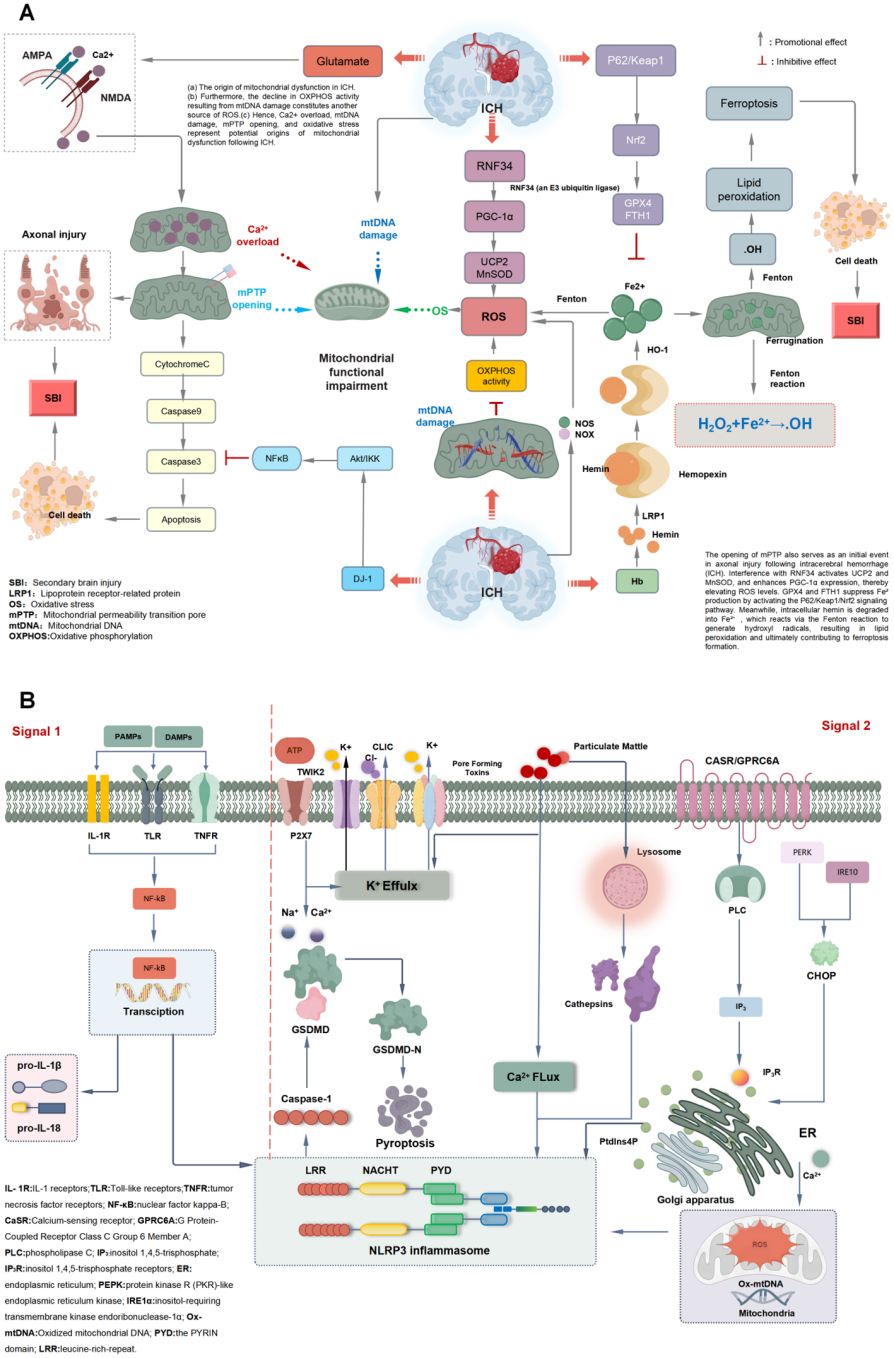
**(A)** Activation of AMPA and NMDA receptors induces mitochondrial Ca²^+^overload, subsequently triggering mPTP opening, activating the caspase cascade, and ultimately leading to cell death. **(B)** Mechanisms of Activation and Regulation of the NLRP3 Inflammasome.

Mitochondria play a crucial role in the pathological process of secondary brain injury following ICH. Their functional state directly affects the survival and repair of nerve cells, and profoundly influences the final prognosis and clinical outcome of patients. As the energy factory of cells, mitochondrial dysfunction is the core reason for the energy metabolism failure of damaged brain tissue, and it also constitutes the significant platform for initiating programmed cell death. Therefore, in-depth exploration of the specific pathological mechanisms behind mitochondrial dysfunction is of great guiding significance for finding early and effective intervention targets, and can provide new directions and perspectives for the formulation of clinical treatment strategies. After the occurrence of ICH, mitochondrial function impairment is influenced by multiple complex factors, including the intensification of oxidative stress, the abnormal opening of the mitochondrial permeability transition pore (mPTP), the structural damage of mitochondrial DNA (mtDNA), and the imbalance of intracellular calcium ion (Ca2^+^) homeostasis leading to overload phenomena; the specific interaction mechanism is shown in **Figure 9A** (99).

### Association between inflammasome immune activation and cell pyroptosis regulation after ICH

The NLRP3 inflammasome is a multi-protein complex composed of NLRP3, the adaptor protein ASC, and the effector protein caspase-1. It activates caspase-1, promoting the maturation and release of pro-inflammatory cytokines such as IL-18 and IL-1β, thereby playing a central regulatory role in the innate immune response. The NLRP3 protein is widely expressed in various immune cells such as neutrophils, monocytes, and lymphocytes. Its molecular structure includes the N-terminal PYD domain, the central NACHT domain, and the C-terminal LRR domain. When ASC binds to NLRP3 through its PY domain, the CARD domain is exposed, which recruits the inactive precursor caspase-1 and cleaves it to activate it. The activated caspase-1 can, on the one hand, cleave Gasdermin D (GSDMD) to produce N-terminal fragments that can perforate the cell membrane, and on the other hand, process the precursors of IL-1β and IL-18 to make them mature and release into the extracellular space. This series of processes ultimately triggers pyroptosis (100–103). The Golgi apparatus is considered an important platform for innate immune signal transduction. Various NLRP3 activators can act on the Golgi apparatus, causing fragmentation of the reverse Golgi network (TGN) and the formation of dispersed dTGN vesicles. The multi-basic regions on the NLRP3 protein can specifically recognize and bind to phosphatidylinositol-4-phosphate (PtdIns4P) on the dTGN membrane, thereby recruiting NLRP3 to the surface of the dTGN membrane. Subsequently, a molecular connection forms between dTGN and ASC, which helps in the assembly and activation of the NLRP3 inflammasome. Previous studies have suggested that NLRP3 can also be recruited and activated in the mitochondrial-associated endoplasmic reticulum membrane (MAM) domain. Recent research has indicated that the protein kinase D (PKD) located in the Golgi apparatus can phosphorylate NLRP3, causing it to dissociate from the MAM and be transported to the Golgi region, thereby fully activating the inflammasome; conversely, inhibiting the activity of PKD will block the activation of the inflammasome. Although the specific role of the TGN in the initiation of the inflammasome remains controversial, and some studies have indicated that the activation of IKKβ alone is sufficient to recruit NLRP3 and trigger inflammasome assembly, research evidence suggests that the Golgi apparatus plays an indispensable role in integrating upstream signals and promoting the localization and activation of NLRP3. **Figure 9B** summarizes the basic structure of the NLRP3 inflammasome and its core molecular mechanisms of initiation and activation (104–106).

### Advances and uncertainties in acute therapy

**Figure 10:**
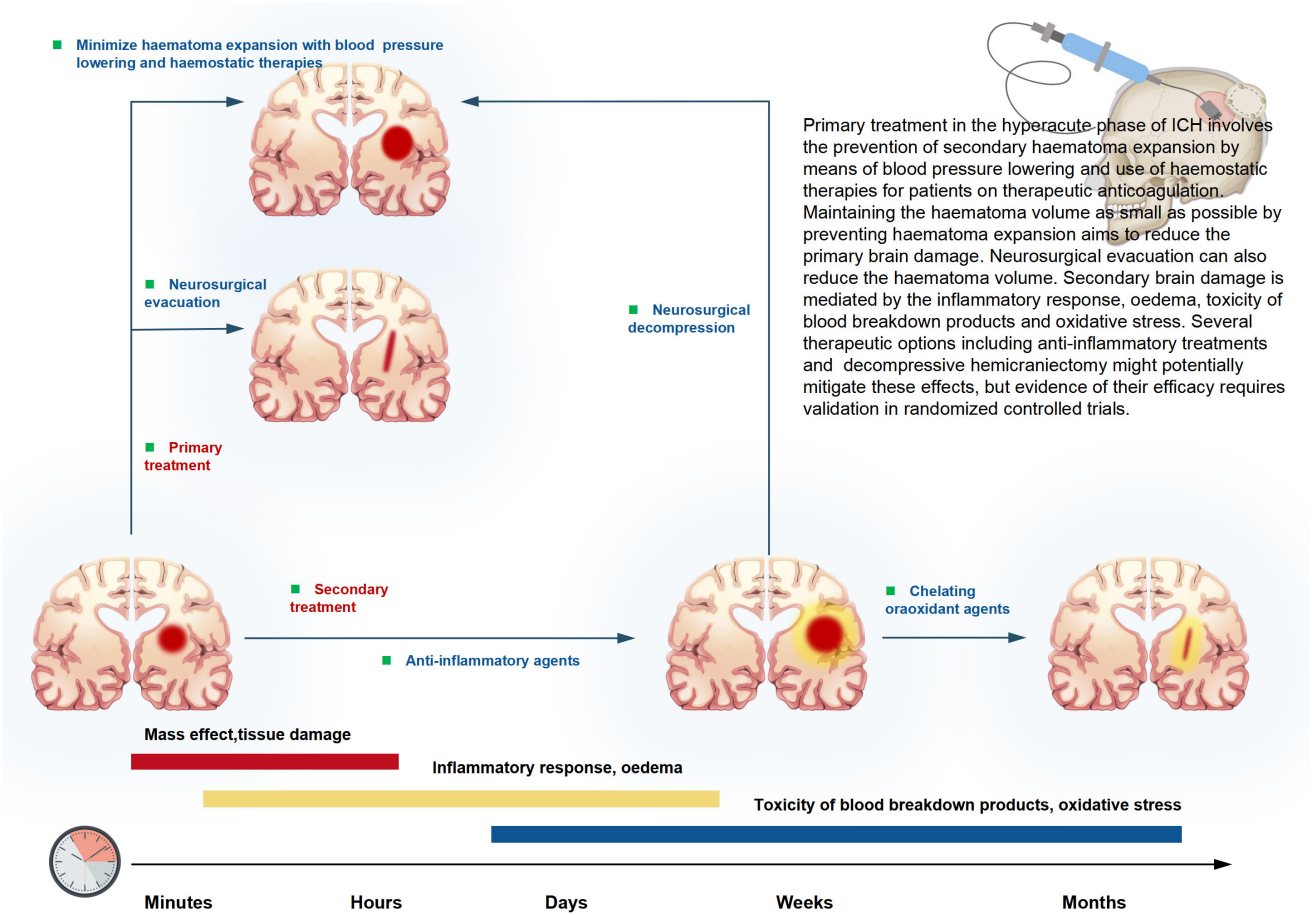
Treatment targets and brain damage after ICH.

ICH is caused by the rupture of blood vessels and the infiltration of blood into the brain parenchyma. The potential triggering factors for vascular rupture in patients with ICH can be classified into two major categories:a sudden and significant increase in blood pressure (107, 108) and infection (109, 110). More specifically, a sudden and significant increase in blood pressure is highly likely to trigger ICH by causing resistance in the walls of fragile small blood vessels; while infection is considered to induce ICH by inducing endothelial dysfunction and coagulation disorders. When a blood vessel ruptures, ICH directly damages the affected nerves, thereby leading to what is known as “primary” or “mechanical” damage (**Figure 10**). Some patients will continue to bleed in the first few hours after the onset of ICH, causing the hematoma to enlarge. This situation can occur before admission or in 20%-30% of admitted patients (111). The enlargement of the hematoma is usually detected in the re-examination of cranial imaging (112, 113), which may involve the enlargement of the main intracerebral blood clots. The main predictors of hematoma enlargement include the time of onset, with the highest risk occurring within 3–6 hours after onset;typically, patients with a history of anticoagulant therapy have a threefold increased risk of hematoma expansion (114, 115). Hematoma enlargement is closely related to the poor neurological prognosis of patients with ICH. A recent study has shown that potential vascular wall damage affects the dynamic process of hematoma enlargement, and in patients with cerebral aneurysms, continuous and prolonged bleeding is more common. Preventing hematoma enlargement is a key goal in the treatment of acute ICH (**Figure 10**) (116).

### Advances in neuroimaging and diagnostic techniques

**Figure 11:**
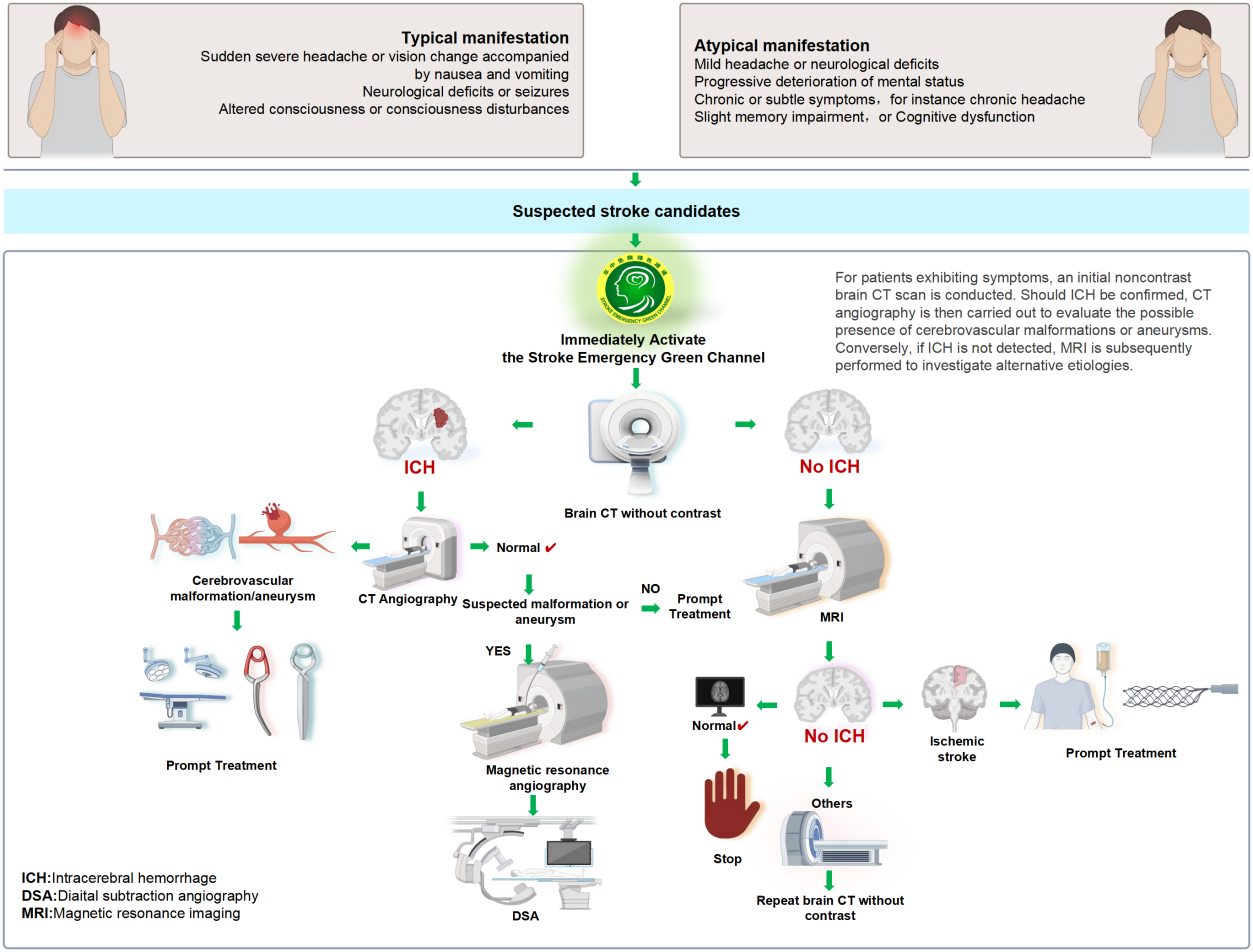
Algorithm for diagnosing ICH.

Although current understanding of the pathophysiological mechanism of ICH is not deep enough to support a comprehensive and efficient treatment system, the rapid progress of neuroimaging technology has significantly improved the accuracy and reliability of diagnosis, assessment, and clinical management of this disease. Among them, hypertensive ICH is widely considered to be closely related to the poor clinical prognosis of patients with ICH. Although the existing treatment methods for HICH still have limitations in terms of effectiveness, using imaging methods for early risk stratification can directly affect the clinical triage decisions and the intensity of subsequent monitoring of patients, thereby providing potential possibilities for improving their final prognosis (117). Therefore, conducting imaging examinations in the early stage of the disease course is an extremely important and indispensable part of the overall management of patients with ICH. The clinical diagnostic process of ICH is shown in **Figure 11** (118).

### Therapeutic targets

**Figure 12:**
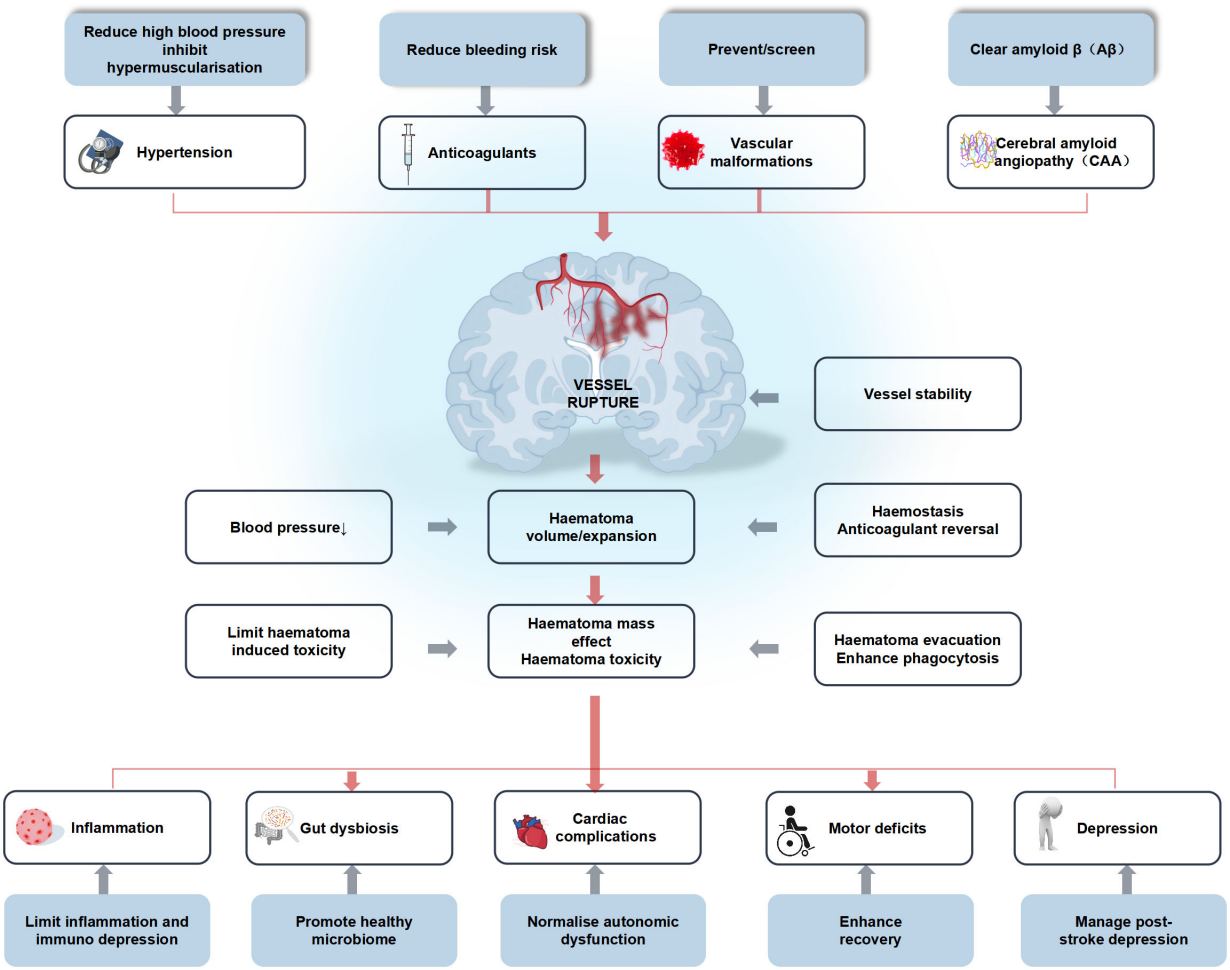
Therapeutic and approaches in ICH.

Over the past two decades, the academic and clinical communities have gradually reached a consensus: ICH, as a unique cerebrovascular disease, has long not received sufficient attention and research regarding its pathophysiological mechanisms and treatment needs. To advance this field, this article systematically summarizes the current main treatment strategies for ICH and outlines the key pathological processes and potential targets that have not been effectively intervened. These summaries aim to provide a clear roadmap for subsequent basic research and clinical translation work (**Figure 12**). Particularly, as research progresses, it is increasingly recognized that ICH is not an isolated event in the central nervous system;other types of cells (such as glial cells, immune cells) and peripheral organs (such as the immune system, coagulation system) are involved in the disease process playing crucial roles. These complex interactions urgently require more in-depth basic research to clarify, and must be fully considered when designing successful treatment intervention plans in the future. This deepening understanding also places higher demands on the experimental tools we use:existing animal models need to be further optimized to more accurately simulate the etiology, dynamic development process, and complete pathological physiological characteristics of human ICH, so that preclinical research results can more reliably guide the development of new therapies targeting the cause and core mechanisms (119).

### Clinical management

**Figure 13:**
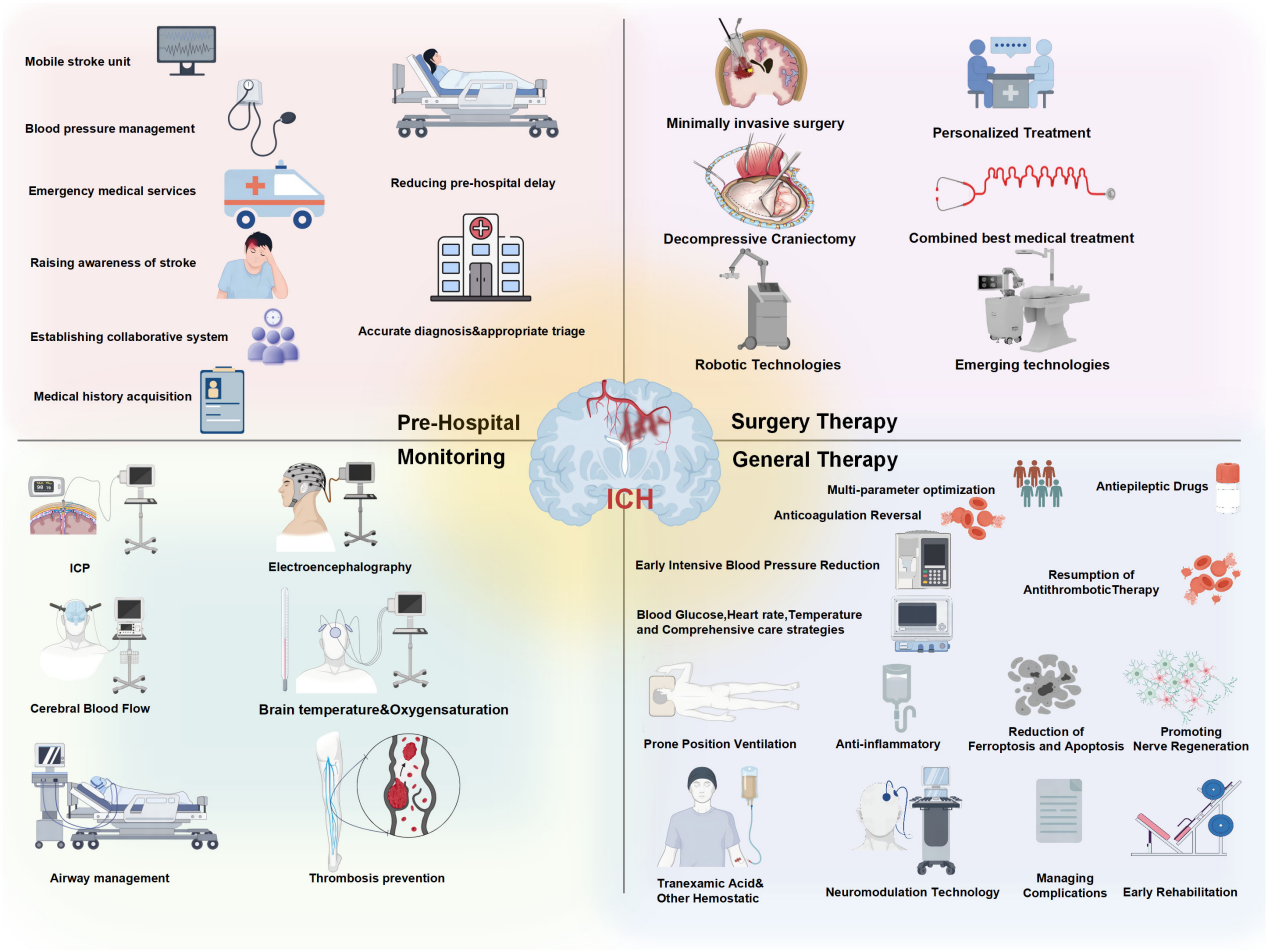
Crucial aspects in the management of ICH.

This section systematically elaborates a series of management measures for patients with acute ICH. These measures cover the entire process of care from pre-hospital emergency to inpatient treatment, aiming to provide patients with timely, standardized, and continuous medical intervention. These plans are specifically designed based on the pathophysiological characteristics and clinical needs of acute ICH. They include the following aspects: pre-hospital emergency and emergency care (120–122); general supportive treatment (reversal of anticoagulation therapy for patients with ICH, re-initiation of antithrombotic treatment, precise management of blood pressure, blood glucose, heart rate, body temperature, and comprehensive care strategies) (123–126); non-surgical treatment (tranexamic acid and other hemostatic agents (127, 128), drug therapy (129, 130)); surgical treatment (131); neuro-intensive monitoring and individualized treatment (132–134); elays in diagnosis and challenges (135–139) (**Figure 13**).

### Neural repair and recovery

Although timely and effective interventions were carried out in clinical settings, most patients with ICH still experience varying degrees of persistent functional impairments such as sensation and movement disorders during the subsequent rehabilitation process. However, the fundamental cause lies in the fact that when ICH occurs, the key neuronal groups responsible for precisely regulating various motor and cognitive functions suffer direct and extensive damage. This damage not only instantly destroys the local neural tissue structure and cellular microenvironment, but also, through complex cascading effects, rapidly spreads and interferes with the dynamic balance and information integration capabilities of the entire brain functional network. Particularly, acute hemorrhage and its subsequent pathological physiological processes may trigger a series of chain-like malignant reactions, such as the massive apoptosis of neurons due to ischemia, hypoxia, and toxic substances, the functional impairment and loss of key synaptic structures that maintain information transmission, and the severe interruption or inhibition of the intrinsic self-repair and regeneration mechanisms of neurons. Once the functional units at the core nodes of the neural network are damaged, the originally precise, orderly, and efficient functional collaboration system of the brain will subsequently become disordered and disconnected, with a significant decline in information processing and output capabilities, ultimately leading to varying degrees of functional decline in multiple aspects such as motor coordination, sensation perception, and advanced cognition.

**Figure 14:**
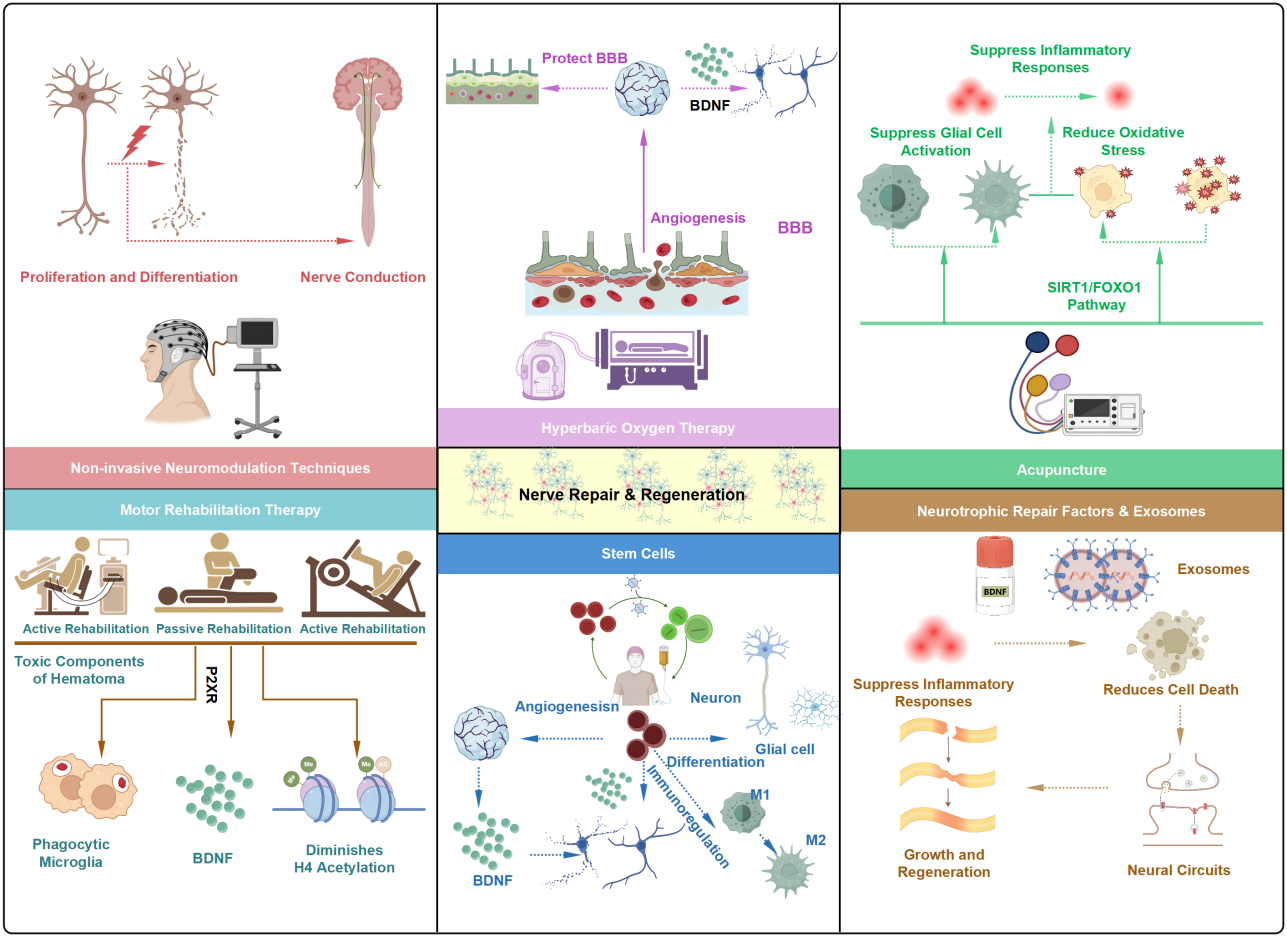
Strategies for neural regeneration and functional restoration in ICH.

However, cutting-edge medical research has brought new hope and light to this predicament:through scientifically implementing a series of highly targeted neuroprotective and repair measures, the damaged neurons and their supporting microenvironment can be repaired and reshaped to a certain extent. These intervention methods aim to alleviate secondary damage, activate the endogenous repair potential, and actively promote the compensatory reconstruction of new neuronal connections and functional neural networks, thereby laying a biological foundation for the gradual recovery of patients’ neurological functions. In order to effectively improve the long-term functional prognosis of patients with ICH and systematically, stage-by-stage promote the maximum effective rehabilitation of their neurological functions, this section will comprehensively and deeply explore the comprehensive neuro-repair strategy system after ICH occurs. These strategies are a multi-dimensional, multi-target intervention set, covering from basic drug treatment, cutting-edge cell and biological therapy, to systematic and individualized rehabilitation training, as well as various evidence-based physical treatment methods, specifically including stem cell therapy (140–144) and endogenous neurogenesis regulation (145–149), the application of neurotrophic factors and exosomes and other biological agents (150–154), task-oriented rehabilitation training (155–159), and the modern application of traditional medical means such as acupuncture therapy (160–162) (**Figure 14**).

### Minocycline in ICH: mechanisms and clinical application

Currently, researchers have fully recognized that the neuroinflammation triggered by ICH plays a crucial role in promoting the progression of secondary brain injury in patients. This realization has prompted numerous studies to focus on finding drugs that can effectively inhibit such harmful inflammation, aiming to block the deterioration pathway of secondary injury at its source and provide new intervention targets for clinical treatment (163). Therefore, precise regulation of neuroinflammation, especially exploring its intrinsic molecular mechanisms and effective intervention strategies, has become an important and continuously focused research direction in this field.

**Figure 15:**
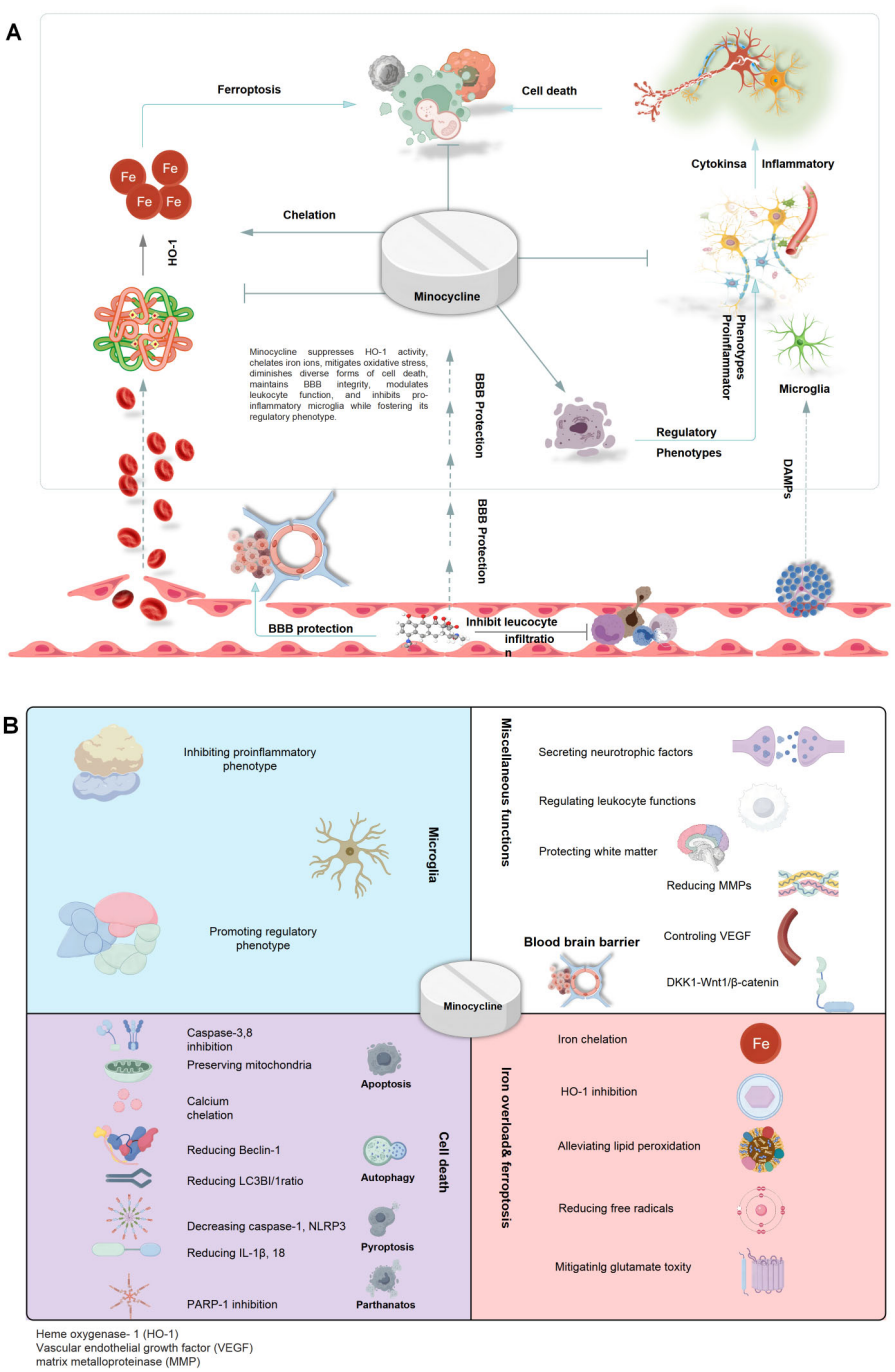
**(A)** Preclinical studies have revealed that minocycline can exert neuroprotective effects by intervening at multiple stages of secondary brain injury induced by ICH. **(B)** The neuroprotective effects of minocycline in experimental ICH are due to multiple mechanisms.

Minocycline has a wide and diverse pharmacological mechanism, providing a solid theoretical basis for its application in the treatment of ICH and indicating its potential clinical value (164, 165). Studies have shown that minocycline can effectively alleviate secondary brain injury after ICH through multiple pathways. Its core mechanism includes: effectively antagonizing iron-induced neurotoxicity and significantly reducing ferroptosis, a form of cell death; enhancing the stability of the BBB and reducing the infiltration of harmful substances into the brain tissue. At the same time, minocycline shows clear inhibitory effects on various programmed cell death pathways, such as apoptosis, autophagy, and pyroptosis. Moreover, it can effectively inhibit excessive activation of microglia, thereby reducing neuroinflammatory responses. These synergistic effects form the basis of its neuroprotective effect (166–169) (**Figure 15A**).

Overall, minocycline, with its unique iron chelating ability, inhibition of the ferroptosis process, and regulation of heme oxygenase-1 (HO-1) activity, can effectively alleviate brain tissue damage related to iron metabolism abnormalities. The molecular mechanisms of these synergistic effects have been fully verified and demonstrated in various experimental conditions, including the bleeding model induced by direct injection of iron ions into the brain (170) (**Figures 15B**).

Although minocycline has been used clinically for many years, it still shows promising efficacy in the treatment of ICH. Research results based on animal experimental models have confirmed that this drug can effectively alleviate neuropathological damage caused by ICH and improve neurological prognosis. This positive effect is mainly attributed to its multiple mechanisms: as mentioned earlier, minocycline can antagonize a series of harmful pathological events that occur after ICH; at the same time, in the ICH model, this drug can take effect quickly and intervene in the disease process in a timely manner. Based on these evidence, we believe that if large-scale clinical trials are conducted in the patient population with ICH in the future, exploring the use of higher concentrations of minocycline in the early stage of the disease and initiating treatment promptly (for example, combined with surgical methods such as hematoma removal), for the functional rehabilitation needs of currently unmet patients with ICH, it is undoubtedly a promising treatment approach.

### Challenges in therapeutics and translation

Although there are still many challenges in the diagnosis and treatment of ICH, the successful translation of laboratory research results based on biomarkers and multi-omics methods into clinical practice is crucial for improving the accuracy of disease diagnosis, clarifying the classification of causes, and achieving refined disease stratification. Among these, achieving early pre-hospital diagnosis, accurate and timely assessment of the condition, and conducting ultra-early intervention are key steps to improving patient prognosis. Therefore, identifying and pre-solving potential delays in pre-hospital transportation and in-hospital treatment, as well as actively exploring the effective integration of new technologies such as artificial intelligence-assisted diagnosis into pre-hospital emergency and care plans, should become the current and future research priorities and preferred directions.

The surgical treatment of ICH is a complex decision-making process that requires a comprehensive consideration of multiple key factors such as surgical techniques, surgical timing, patient selection, adjunctive treatments, and the accessibility of medical resources. The future research direction should focus on exploring the integration of minimally invasive surgery, innovative drug therapies, and advanced robotic technologies to achieve the most optimal and individualized treatment goals for patients with ICH. Based on precise grasping of the surgical intervention window and reasonable selection of surgical methods, two strategies are believed to have the potential to reduce acute-stage damage by promoting local hemostasis: one is to utilize the synergistic effect of drugs to enhance hemostatic efficacy, and the other is to perform precise local drug delivery through the surgical channel. However, the effectiveness and safety of these methods still need to be confirmed through rigorous clinical trials in the future.

Currently, there is a lack of widely recognized best practice standards in the field of continuous monitoring of ICH patients. How to accurately interpret data from various brain monitoring devices (such as intracranial pressure, cerebral blood flow, electroencephalogram, etc.) and how to effectively convert this information into clinical treatment decisions remain issues that need to be clarified. Developing a comprehensive monitoring system that integrates multiple monitoring parameters is expected to present a more complete picture of the brain’s function and pathological state, thereby providing more precise and reliable basis for treatment intervention.

According to statistics, approximately 5% of patients with cerebral hemorrhage will experience recurrence of the condition each year (171, 172). Although the academic community generally agrees that strict blood pressure control and management may have a greater value in preventing secondary ICH than in preventing ischemic stroke, there are still several unresolved core issues. For example, will overly strict blood pressure control strategies pose potential risks to important organs such as the heart and kidneys? Should the blood pressure control targets for patients with ICH due to different causes or bleeding sites be different? At present, there are no clear conclusions on these issues. Additionally, for patients with atrial fibrillation or those who need to take antithrombotic drugs for a long time, when is the most appropriate time to resume anticoagulation therapy, and what are the safety profiles of different anticoagulation regimens, still require more randomized controlled trials to provide high-level evidence. Similarly, such healthy lifestyle practices as quitting smoking, limiting alcohol consumption, controlling blood sugar, adhering to regular exercise, and adopting a low-salt diet for the prevention of secondary ICH can bring what degree of benefit to the secondary prevention of cerebral hemorrhage, also requires more research data to support and clarify.

## Limitations of current research

### Limitations of current experimental ICH models

#### Differences between animal models and human pathophysiology

Species-specific differences in cerebral vascular anatomy: Human cerebral hemorrhage occurs mostly in the basal ganglia region, supplied by the choroidal artery and other vessels. The vascular network of rodents is simple and lacks collateral circulation, resulting in deviations from human clinical conditions. Age and comorbidity factors: Experiments mostly use young, healthy male animals, while clinical patients are mostly elderly and have underlying diseases. The pathological process of the model cannot accurately reflect the condition of elderly patients, and the efficacy of drug intervention may be overestimated (173, 174).

#### Limitations of model establishment methods

Collagenase injection model: Injecting bacterial collagenase induces hemorrhage, which is simple to operate and the size of the hematoma is easy to control. However, the introduction of exogenous collagenase triggers an inflammatory response, which does not match the sterile inflammatory mechanism in clinical practice, and it is difficult to simulate the dynamic process of hematoma expansion (175–178). Autologous blood injection model: Extracting autologous blood and injecting it into the brain parenchyma, the composition of the hematoma is similar to that of humans, but after blood injection, the hematoma quickly solidifies, and the injection pressure is difficult to control, insufficiently simulating the coagulation cascade reaction. Microsphere or balloon compression model: Simulating the effect of a space-occupying lesion, simplifying the pathological process of cerebral hemorrhage, ignoring the neurotoxic reactions caused by blood components, and unable to study the toxic effects of the hematoma (179–181). Limitations of clinical outcome assessment.

Subjectivity of neurological function scoring: Common scoring methods focus on motor function and lack assessment of non-motor functions, making it difficult to simulate the long-term symptoms of clinical patients. Bias in acute phase assessment: Most studies focus on the acute phase, and key issues such as long-term recovery receive less attention, making it difficult for the effective therapies in animal experiments to improve long-term prognosis. Lack of standardized imaging assessment: Clinically, CT and MRI are relied upon, while laboratory estimation of hematoma volume is time-consuming and affected by multiple factors. MRI scans have long scan times and insufficient resolution, making it difficult to conduct dynamic and continuous monitoring (182–184).

#### Differences in hematoma composition and clinical conditions

Lack of models related to anticoagulant drugs-induced cerebral hemorrhage: The incidence of anticoagulant-induced cerebral hemorrhage in clinical practice has increased, and existing models are difficult to simulate the bleeding manifestations under anticoagulant conditions. Complexity of hematoma composition: Clinical hematoma compositions are complex, and the injected blood in the experimental model quickly coagulates, the degradation product spectrum is different from that in clinical practice, and the kinetics of iron ion release may be inconsistent (185–187).

#### Underestimation of gender and genetic factors

Dominance of male animals: Due to concerns about the interference of female animals’ hormonal cycles with the experimental results, approximately 80% of cerebral hemorrhage studies only use male animals. However, the incidence and severity of ICH in females (especially after menopause) differ from those in males, and female animals respond differently to neuroprotective agents, resulting in poor extrapolation of experimental results to clinical outcomes. Single genetic background: Laboratory commonly uses inbred animal strains, with highly uniform genetic backgrounds. However, ICH is a complex polygenic disease related to hypertension and vascular fragility, and the genetic backgrounds of different races have significant effects on the prognosis of ICH. The current models are difficult to simulate the heterogeneity of genetic influences (188–190).

#### Gap in clinical translation

Manually set treatment window: Intervention measures in experimental models are usually implemented within 1–6 hours after the occurrence of ICH, which is difficult to achieve in clinical practice. The delayed treatment of clinical ICH makes the effective early intervention measures in animal experiments ineffective in clinical trials (191).

Lack of multiple comorbidity models: Most ICH patients have diseases such as hypertension and heart disease and often take medications. Current experimental models cannot simulate the coexisting conditions, leading to overly optimistic or one-sided evaluations of the effectiveness of drugs in a complex clinical environment (192).

Difference in hematoma clearance methods: Clinically, large-area or intraventricular hemorrhage is often surgically cleared, while animal models mostly use direct aspiration or drug-induced dissolution, lacking simulation of surgical trauma, infection risks, and postoperative edema rebound, resulting in a significant gap from the clinical surgical scenario (193, 194).

Although we have made the greatest efforts to comprehensively and systematically summarize and generalize the pathological mechanism and clinical treatment strategies of ICH, due to the limitations of the article’s length, it is indeed difficult to elaborate on every relevant aspect in detail and depth. Especially regarding the specific mechanism of peripheral immune cells after ICH, given that it involves a large number of complex and interwoven cell signal transduction pathways and networks, we cannot conduct a detailed discussion and analysis in this article. Looking forward to the future, related research should attempt to adopt a broader and more integrated perspective, and consider including other types of intracranial hemorrhage situations for examination, in order to provide more comprehensive and more inspiring reference basis for the application transformation in clinical practice and the subsequent deeper exploration of mechanisms.

## Conclusions and future perspectives

ICH is a medical challenge of great severity in clinical practice due to its extremely high mortality and disability rates. To effectively address these challenges, continuous and in-depth scientific research should be carried out, and more targeted personalized treatment plans should be developed. This is of great significance for reducing the social and economic burden caused by ICH, improving the prognosis of patients, and enhancing their long-term quality of life.

For patients with acute ICH, the timing of initiating hemostasis treatment is crucial. It should be carried out promptly within the golden window period of the first few hours after the onset of the disease. Therefore, initiating hemostasis intervention as early as possible through advanced technologies such as pre-hospital emergency systems and mobile surgical units can maximize the rescue of dying brain tissue and significantly improve the degree of neurological function recovery and the possibility of returning to society in the future. On this basis, personalized and precise adjustments to clinical treatment plans will further optimize the diagnostic process and treatment strategies for this disease.

However, accurately and promptly determining the optimal intervention time window for each patient with ICH has always been a complex and arduous task. This requires close collaboration and cross-fusion of multiple disciplines such as neuroimaging, molecular biology, symptomatology, and bioinformatics, combined with the assistance of artificial intelligence, through the integration of multi-dimensional information, to more accurately predict the trajectory of hematoma expansion, secondary damage, and neurological function outcome after ICH.

Fortunately, the continuous emergence of preclinical basic research on ICH and the gradually advancing clinical trials are injecting new impetus into revealing its pathological mechanisms and exploring new therapies, and bringing continuous hope for ultimately reducing the overall burden of this devastating disease.

### Future directions

The immune response after ICH is highly heterogeneous, with this heterogeneity manifested not only in individual differences but also in functional variations among different brain regions and different immune cell subsets. In the future, high-resolution technologies such as single-cell RNA sequencing and spatial transcriptomics should be utilized to construct a complete immune cell map of ICH. The research should focus on identifying key molecular switches that drive neurotoxic (such as pro-inflammatory microglia and neutrophils) and neuroprotective (such as regulatory T cells and repair-type macrophages) immune phenotypes. Clarifying the sources of this heterogeneity (such as genetic background, bleeding site, and differences in hematoma composition) will lay the foundation for achieving precise immune intervention, aiming to suppress harmful immune responses while maintaining or enhancing beneficial reparative immune activities. Regarding aging and comorbidity models, currently, most clinical patients with ICH are elderly and often have comorbidities such as hypertension and diabetes. However, most current basic research is based on young, healthy animal models, which severely limits the translational value of the research results. Future research must prioritize the establishment of more realistic complex models, such as inducing ICH in aged animals or those with comorbidities such as hypertension, diabetes, and chronic kidney disease. Exploring how aging and comorbidity states specifically reshape the immune response after ICH, including functional changes in immune cells (immunosenescence), aging of the BBB, and systemic inflammatory environments. Using these models for drug screening and mechanism validation is a key step in discovering effective treatment methods applicable to real patient populations. The ultimate goal of ICH research is clinical translation. In the future, specific directions should be focused on: 1) Identifying biomarkers: through cerebrospinal fluid or peripheral blood, identifying core molecules or cell markers that can predict the immune status, edema evolution, and neurological prognosis after ICH to guide clinical stratified treatment. 2) Exploring targeted delivery: overcoming the BBB and developing nanomedicines or biological agents targeting specific immune targets. 3) Designing preclinical-clinical bridging trials: based on these superior animal models (such as aged comorbidity models) and reliable biomarkers, designing a translational strategy from the bedside to the laboratory and back to the bedside. For example, using biomarkers to screen patient subgroups that may benefit from specific immune regulatory treatments and conducting precise, small-sample confirmatory clinical trials.
